# Sponge diversification in marine lakes: Implications for phylogeography and population genomic studies on sponges

**DOI:** 10.1002/ece3.9945

**Published:** 2023-04-13

**Authors:** Diede L. Maas, Stefan Prost, Christiaan A. de Leeuw, Ke Bi, Lydia L. Smith, Purwanto Purwanto, Ludi P. Aji, Ricardo F. Tapilatu, Rosemary G. Gillespie, Leontine E. Becking

**Affiliations:** ^1^ Marine Animal Ecology Wageningen University & Research Wageningen The Netherlands; ^2^ LOEWE Centre for Translational Biodiversity Genomics Senckenberg Natural History Museum Frankfurt am Main Germany; ^3^ South African National Biodiversity Institute National Zoological Gardens of South Africa Pretoria South Africa; ^4^ Museum of Vertebrate Zoology University of California Berkeley Berkeley California USA; ^5^ Computational Genomics Resource Laboratory, California Institute for Quantitative Biosciences University of California Berkeley Berkeley California USA; ^6^ Coral Triangle Center Bali Indonesia; ^7^ Research Centre for Oceanography, Indonesian Institute of Sciences Lembaga Ilmu Pengetahuan Indonesia Jakarta Indonesia; ^8^ Marine Science and Fisheries Departments and Research Center of Pacific Marine Resources State University of Papua Manokwari Indonesia; ^9^ Department of Environmental Science, Policy and Management University of California Berkeley Berkeley California USA; ^10^ Aquaculture and Fisheries, Naturalis Biodiversity Center Wageningen University & Research Wageningen The Netherlands

**Keywords:** genetic resolution, marine biodiversity, Porifera, RADseq, seascape genomics, *Suberites diversicolor*

## Abstract

The relative influence of geography, currents, and environment on gene flow within sessile marine species remains an open question. Detecting subtle genetic differentiation at small scales is challenging in benthic populations due to large effective population sizes, general lack of resolution in genetic markers, and because barriers to dispersal often remain elusive. Marine lakes can circumvent confounding factors by providing discrete and replicated ecosystems. Using high‐resolution double digest restriction‐site‐associated DNA sequencing (4826 Single Nucleotide Polymorphisms, SNPs), we genotyped populations of the sponge *Suberites diversicolor* (*n* = 125) to test the relative importance of spatial scales (1–1400 km), local environmental conditions, and permeability of seascape barriers in shaping population genomic structure. With the SNP dataset, we show strong intralineage population structure, even at scales <10 km (average *F*_ST_ = 0.63), which was not detected previously using single markers. Most variation was explained by differentiation between populations (AMOVA: 48.8%) with signatures of population size declines and bottlenecks per lake. Although the populations were strongly structured, we did not detect significant effects of geographic distance, local environments, or degree of connection to the sea on population structure, suggesting mechanisms such as founder events with subsequent priority effects may be at play. We show that the inclusion of morphologically cryptic lineages that can be detected with the COI marker can reduce the obtained SNP set by around 90%. Future work on sponge genomics should confirm that only one lineage is included. Our results call for a reassessment of poorly dispersing benthic organisms that were previously assumed to be highly connected based on low‐resolution markers.

## Graphical abstract

High‐resolution genotyping of the marine sponge *Suberites diversicolor* from marine lakes confirms genetic lineages, but provides new evidence for strong intralineage population structure. Geographic distance and local environments did not explain genetic differentiation, suggesting other mechanisms such as founder events with priority effects may be at play. Results indicate the potential need to reassess panmictic assumptions from poorly dispersing benthic organisms.

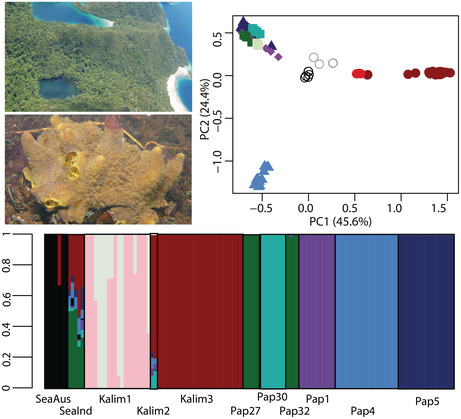

## 1 INTRODUCTION

Isolating mechanisms causing population genomic structuring still remain elusive for many marine organisms (Liggins et al., 2013; Peijnenburg & Goetze, 2013; Selkoe et al., 2016). Examples of such mechanisms are dispersal limitation due to geographic distance resulting in isolation‐by‐distance patterns (Chaves‐Fonnegra et al., 2015; Pérez‐Portela et al., 2015; Wright, 1943), and dispersal/establishment limitation due to ecologically heterogeneous habitats resulting in a pattern of isolation‐by‐environment (Giles et al., 2015; Nosil et al., 2009; Orsini et al., 2013; Rundle & Nosil, 2005). In some cases, patterns of genomic structure in marine organisms may not be clearly linked to geographic or environmental influences (Cornwell et al., 2016; Miller et al., 2018; Taboada et al., 2018). Here, other explanations may include other barriers to dispersal, such as ocean currents, resulting in an isolation‐by‐resistance pattern (McRae, 2006), or processes involving historical contingency (Fukami, 2015). Recent work on benthic invertebrates has shown strong population genomic structure, providing evidence against the previously widely held belief that marine organisms are panmictic due to few barriers to dispersal (e.g., Bierne et al., 2016; Marshall et al., 2010; Van Wyngaarden et al., 2017). Understanding population structure at smaller scales is relevant as marine sessile invertebrates can show ecological dynamics and incipient speciation at these scales (<500 km, Bernatchez et al., 2019; Van Wyngaarden et al., 2017; Xuereb et al., 2018). It is also the scale at which human activities can cause direct change, for example, through habitat change and degradation, and through restoration activities and marine spatial planning.

In marine invertebrates, which encompass the largest diversity in the marine animal kingdom (Chen, 2021), there are still outstanding questions on the processes that lead to the small‐scale structuring of population genomic diversity. A key issue is that for many nonmodel marine invertebrate species, there is still a dearth of high‐resolution genomic approaches that allow detection of small‐scale population genomic structure, diversity, and demographic histories (Oleksiak & Rajora, 2020). Sponges are a prime example in this regard. Sponges are integral yet often underappreciated assets of benthic communities (Bell, 2008; De Goeij et al., 2013; Webster & Thomas, 2016). Given that sponges are generally considered to be poor dispersers as their larvae have limited swimming capacity and are short‐lived (Maldonado, 2006), patterns of strong genetic divergence over relatively small geographic ranges would be expected. However, studies so far typically find connectivity at scales of 100s–1000s of kilometers (e.g., De Bakker et al., 2016; Taboada et al., 2018). Studies that have sought to understand the processes shaping genetic structure in sponges have revealed species complexes with divergence among morphologically cryptic lineages (Pérez‐Portela & Riesgo, 2020; Uriz & Turon, 2012; van Oppen et al., 2002), yet seem to show little genetic diversity and signatures of panmixia within species encompassing large geographic areas in shallow (de Bakker et al., 2016; Whalan et al., 2008) and in deep sea areas (Ekins et al., 2016; Taboada et al., 2018). The findings of high connectivity within species may indicate that the dispersal ability of sponges is inherently greater than that of other marine organisms, with potential explanations including rafting, asexual budding, and sperm‐mediated gene flow (DeBiasse et al., 2014; Maldonado & Uriz, 1999; Wörheide et al., 2008). However, this runs counter to sponges generally being considered to be poor dispersers with short‐lived larval stages with limited swimming capacity (Maldonado, 2006).

The lack of structure that has been found in sponge populations may be the result of the dearth of high‐resolution genomic studies (Oleksiak & Rajora, 2020; Pérez‐Portela & Riesgo, 2020). The majority of studies on sponge phylogeography and population structure have deployed mitochondrial markers (mtDNA) such as Cytochrome *c* oxidase I (*COI*) and ATP6, and nuclear markers such as introns, internal transcribed spacers (*ITS*) (Wörheide et al., 2008) and microsatellites (Pérez‐Portela & Riesgo, 2020; Uriz & Turon, 2012; Van Oppen et al., 2002). Although widely used in phylogeographic and population genetic studies (Avise, 2000, 2009), mitochondrial markers exhibit low mutation rates in sponges (Huang et al., 2008; Wörheide et al., 2005), resulting in lower diversity. In contrast, studies using *ITS* markers have shown more structure (Becking et al., 2013; Bentlage & Wörheide, 2007; Ekins et al., 2016), but generally at large spatial scales (~1000 km). Furthermore, *ITS* markers can be hampered by intragenomic polymorphisms (Frankham et al., 2002; Wörheide et al., 2004), clouding patterns of true population structure. Finally, microsatellites could be reliable and sufficiently variable to detect population structure (Taboada et al., 2018), yet are time‐consuming to design de novo for each species (Frankham et al., 2002; Pérez‐Portela & Riesgo, 2020), can be confounded by homogenizing forces of evolution (Van Oppen et al., 2002), and generally relatively few are used per study (<20). An increase in the number of molecular markers is expected to advance inferences on structure and demography (Allendorf et al., 2010; Kelley et al., 2016; Pérez‐Portela & Riesgo, 2020), allowing researchers to reassess assumptions of panmixia within sponge lineages.

Recently, there has been an increase in the use of reduced representation genomic methods and Single Nucleotide Polymorphisms (SNPs) for population genomic studies on nonmodel organisms (e.g., Baird et al., 2008; Catchen et al., 2017; Peterson et al., 2012; Puritz et al., 2014). The additional power of an increased marker panel has been demonstrated in for example mussels (Becking et al., 2016; de Leeuw et al., 2020; Maas et al., 2018), fish (Bradbury et al., 2015; D'Aloia et al., 2020; Lemopoulos et al., 2019; Sunde et al., 2020), and sponges (Taboada et al., 2022). However, high‐resolution studies on sponges are lagging behind (Pérez‐Portela & Riesgo, 2020), with the notable exception by Brown et al. (2014), Brown et al. (2017), Leiva et al. (2019), and Taboada et al. (2022). Using restriction site‐associated DNA sequencing (RADseq), techniques such as ddRAD (double digest RADseq, Peterson et al., 2012), such as in Leiva et al. (2019) and Taboada et al. (2022), may increase the number of retained SNPs to thousands and provide the necessary resolution. However, most of these studies still used a limited marker panel and showed no differentiation at scales below 100 km. In this study, we used data with >4000 SNPs to investigate population structuring and demography in a shallow water sessile marine invertebrate in relation to geographic distance, permeability of barriers, and environmental variables using marine lake ecosystems.

Islands and other insular systems provide ideal models to test factors that underlie population structure since they are well‐defined and are of lower complexity than open areas (Warren et al., 2015). Marine lakes are insular bodies of seawater surrounded completely by land that maintain connection with the surrounding sea through caves or porous rock (Becking et al., 2011; Dawson et al., 2009; Hamner et al., 1982; Holthuis, 1973). The extent of the connection of marine lakes to the sea ranges from being highly connected where seawater moves in and out of the lake through caves, to highly isolated where seawater has to travel through porous rock. Marine lakes are common in Vietnam, Palau, and Indonesia, particularly in East Kalimantan and in West Papua (Becking et al., 2011, 2015; Dawson et al., 2009). Having originated roughly at the same time (after the Last Glacial Maximum approximately 8000–10,000 years ago; Sathiamurthy & Voris, 2006; Tomascik & Mah, 1994), marine lakes represent relatively controlled biotopes where each lake can be seen as an independent replicate of eco‐evolutionary dynamics. Although marine lakes have barriers to connectivity and may represent local environments distinct from open marine systems, they could still be representative of heterogenous coasts that are prevalent in the Indo‐Pacific. Furthermore, studying marine lakes can help in better understanding drivers of genomic structure such as founder effects, and bottlenecks and expansion events in marine populations.

Sponges are usually well‐represented in marine lakes, both in diversity and in biomass (Azzini et al., 2007; Becking et al., 2011, 2013; Cleary et al., 2013). The sponge *Suberites diversicolor* (Porifera, Demospongiae, Suberitidae, Becking & Lim, 2009) has been found to occur in Indonesian marine lakes and brackish coastal areas (Becking & Lim, 2009; Cleary et al., 2013). Using *COI* and *ITS* genetic markers, Becking et al. (2013) studied its phylogeography from multiple marine lakes and lagoon populations in the Indo‐Pacific. They identified two distinct genetic lineages (Lineage A and B) and regional structuring, yet did not observe structure at smaller spatial scales. The lack of structure could be explained by recurrent gene flow among lakes or by lack of resolution of genetic markers used by Becking et al. (2013), as they recovered a low number of haplotypes (4 for *ITS* and 3 for *COI*). Given the high genomic structuring observed in co‐distributed species from marine lakes (de Leeuw et al., 2020; Gotoh et al., 2011; Maas et al., 2018, 2020), we expect that the markers used did not provide sufficient resolution to detect signals.

Selecting nine marine lakes and two lagoon locations in the Indo‐Pacific at different spatial scales (1–1400 km), with different environmental conditions, and along a gradient of connection to the surrounding sea, we assessed the relative influence of these drivers on genomic structure of *S. diversicolor*. Furthermore, we (a) optimized laboratory and bioinformatic filtering methods for low‐coverage RAD‐generated data using old stock DNA extractions to assess effects of filtering, (b) compared phylogeographic and population genetic structure of *COI* and *ITS* markers from Becking et al. (2013) to RAD‐generated markers, and (c) assessed the influence of phylogenetic level on the number of markers retained. We expect populations to be highly structured at small spatial scales, reflecting the life history of benthic sponges with short larval durations, with this structure being detectable via the higher resolution gained via using a RAD approach. Next, if the structure is linked to dispersal potential due to geographic distance, we expect to find isolation‐by‐distance patterns, where gene flow decreases with increasing distance. In contrast, if the environment strongly influences population structure, we expect to find patterns of isolation‐by‐environment, where marine lakes similar in local environmental conditions should resemble each other regardless of the extent of geographic distance. If the extent of connection to the surrounding sea influences dispersal potential, we expect to observe an isolation‐by‐resistance pattern. Here, populations in highly connected lakes would show signals of genomic connectivity among each other and the lagoon populations, whereas isolated marine lakes would be particularly distinct, reflecting low dispersal from the sea. Finally, populations in isolated marine lakes should show evidence for strong genetic bottlenecks, reflecting the ontogeny of the lakes and subsequent low immigration from the sea populations, while highly connected lakes should not, due to high water exchange.

## 2 MATERIALS AND METHODS

### 2.1 Sample collection and lake profiling

**FIGURE 1:**
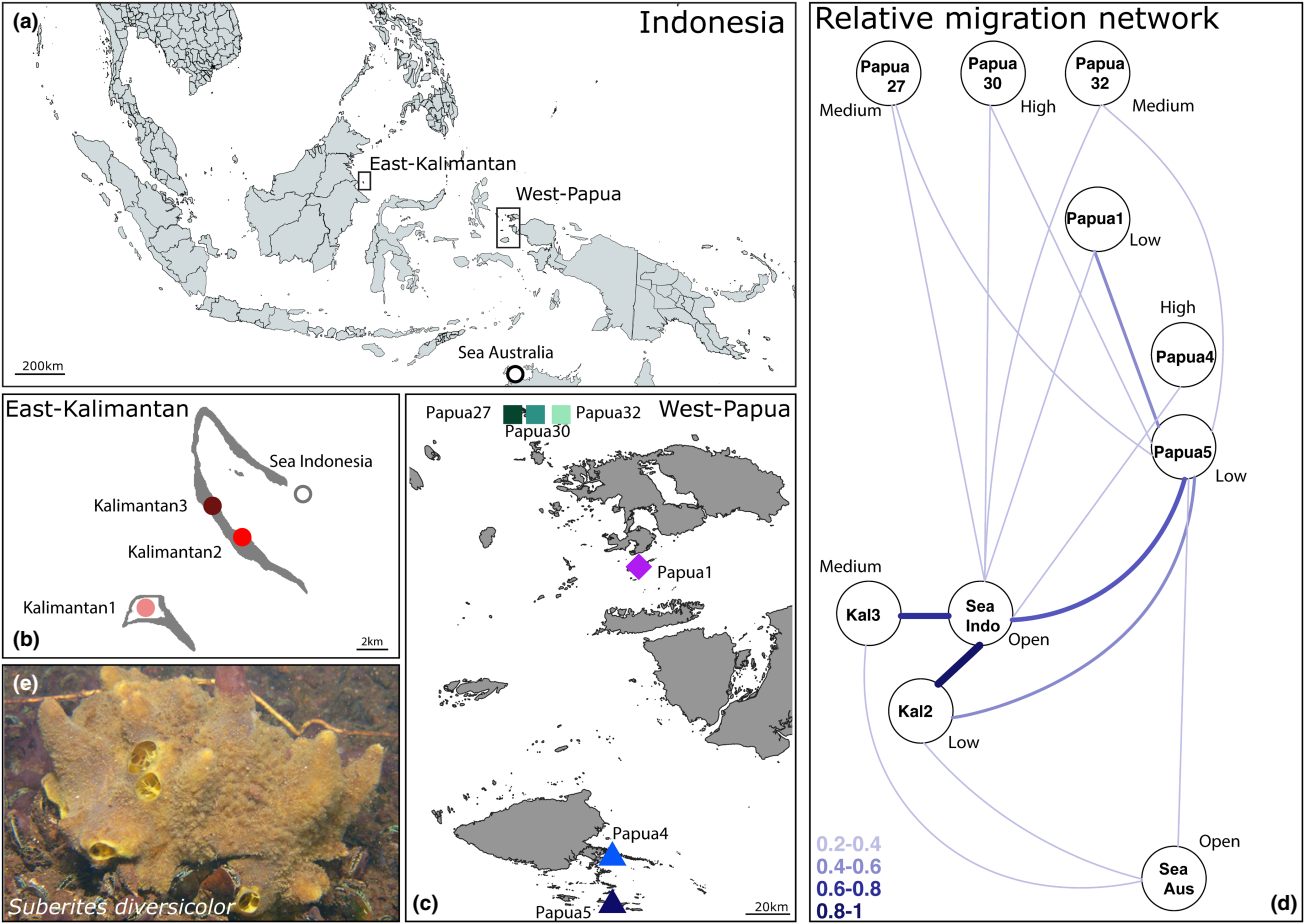
Sampling sites of *Suberites diversicolor* from nine marine lakes and two lagoon locations and associated relative migration networks. (a) Overview of Indonesia including two geographic regions sampled: Berau, East‐Kalimantan and Raja Ampat, West‐Papua. It also shows the location of the Australian lagoon sampled (Sea Australia). (b) Zoom of Berau, with locations of three marine lakes (Kalimantan1, Kalimantan2, and Kalimantan3) and one lagoon (Sea Indonesia). (c) Zoom of Raja Ampat, with locations of six marine lakes (Papua27, Papua30, Papua32, Papua1, Papua4, and Papua5). (d) Relative migration network including only samples from Lineage B based on 4826 SNPs and run with 1000 bootstraps. Fractions of relative migration are displayed and categories of level of connection to the surrounding sea are indicated. (e) Specimen of *S. diversicolor*, photograph by L.E. Becking.

Marine lakes are not common and occur predominantly in Indonesia, Vietnam, and Palau (Dawson et al., 2009). The sponge *Suberites diversicolor* is found in many marine lakes, but some of the sampled lakes had very low densities; therefore, sample sizes were smaller (see Becking et al., 2013 table 2 for densities). As *S. diversicolor* is not frequently found outside of marine lakes, we took an opportunistic sampling strategy. Tissue samples (~1 cm^3^) were collected from 168 individuals of *Suberites diversicolor* (Figure 1 and Table 1). One lagoon was sampled in Darwin, Australia (Sea Australia), one lagoon in East‐Kalimantan (Sea Indonesia). In East‐Kalimantan, we additionally sampled three marine lakes (Kalimantan1, Kalimantan2, and Kalimantan3), and six marine lakes in West‐Papua (Papua27, Papua30, Papua32, Papua1, Papua4, and Papua5). As many locations have no official names, we used a coding system consistent with de Leeuw et al. (2020) and Maas et al. (2018, 2020). Of these locations, nine overlap with the sponge phylogeography study of Becking et al. (2013) (Table S1 for corresponding lake codes), two additional marine lakes were sampled for this study (Papua5 and Papua27). Samples were collected between 1 and 5 m depth while snorkeling, collecting individuals at least 25 m apart. In the field, tissue samples were cleaned of any debris or metazoan symbionts visible to the naked eye, and immediately preserved in 99% ethanol or RNAlater after excision at 0–4°C (4–8 weeks), and upon returning to the laboratory stored in a −20°C freezer until further use.

**TABLE 1:** Overview of sampling in marine lakes and lagoon locations.

Code	Location	Connection category	Fraction tidal amplitude	Area (×1000 m^2^)	Max. depth (m)	Average temperature	Average salinity	Number specimens passed QC	Nucleotide diversity	Heterozygosity (He)
Sea Australia	Australia	Open						7	0.0095	0.117
Indonesia	Region: Island									
Sea Indonesia	Berau	Open				29.0	33.5	5	0.0101	0.157
Kalimantan1	Berau: Kakaban	Low	0.1	4900	12	30.0	23.5	20		
Kalimantan2	Berau: Tanah Banban	Low	0.4	232		29.5	26.0	2	0.0074	0.034
Kalimantan3	Berau: Maratua	Medium	0.5	140	17	29.5	27.0	26	0.0050	0.081
Papua27	Papua: Wayag	Medium		22	2	29.5	31.0	5	0.0037	0.038
Papua30	Papua: Wayag	High	0.8	13	4	32.4	28.9	8	0.0045	0.052
Papua32	Papua: Wayag	Medium	0.5	6	6	31.2	30.7	4	0.0053	0.059
Papua1	Papua: Gam	Low	0.1	89	19	32.3	24.0	11	0.0036	0.054
Papua4	Papua: Misool	High	0.8	14	20	31.7	25.9	19	0.0047	0.080
Papua5	Papua: Misool	Low	0.3	4	5	31.5	28.9	18	0.0060	0.095

*Note*: Recorded are site codes, location, physiographic, environmental, and genomic parameters, and number of individuals retained after filtering. Explanations of how physiographic parameters (lake area, depth and connection) as well as how the local water quality was measured (temperature and salinity) can be found in the methods section. In the calculation of population genomic parameters (nucleotide diversity and heterozygosity) only localities containing samples from Lineage B were considered.

Lake area (m^2^) was approximated using Google Earth Pro (v. 7.3.2), maximum depth was measured using a handheld sonar system (Hawkeye), and water parameters temperature (°C) and salinity (ppt) were measured with a YSI Professional Plus multimeter at 10 locations per lake at 1 m intervals from the surface to 5 m depth. To define the connection to the surrounding sea, we measured maximum tidal amplitude simultaneously in the lake and the sea using Hobo water‐level loggers (Onset HOBO U20L). The degree of water exchange between the marine lakes and the adjacent sea was assessed by placing a water‐level logger inside the marine lake and one directly outside in the surrounding sea during a 48 h period. Pressure (Pa) was converted to depth (m) using the Hoboware Pro 3.7.16 software. The fraction of tidal amplitude of the lake compared with the sea was then calculated by (Lake_max_)/(Sea_max_), where max stands for the maximum water level in either the lake or the sea. A maximum value of 1 would indicate limited (or no) obstruction to water flow in and out of the lake, and the minimum value of 0 would mean there is no water exchange at all. We categorized the level of connection to the surrounding sea as low (≤0.4), medium (0.5–0.7), and high (0.8–1). The sea locations were categorized as open.

### 2.2 DNA extraction, library preparation and sequencing

DNA was extracted using the DNeasy Blood & Tissue kit (Qiagen), with the only modification from manufacturer instructions being an extended lysis time (overnight). DNA quality and quantity were assessed using 1.5% agarose gels and Qubit dsDNA HS assays. Next, double digest restriction site‐associated DNA (ddRAD) libraries were prepared following the protocol of Peterson et al. (2012). The adapted protocol used by the current study can be found in the Appendix S2. In brief, genomic DNA (600 ng) was double‐digested using enzymes SphI‐HF (rare‐cutting) and MlucI (frequent‐cutting) (See Appendix S1 for example of a successful enzyme digestion). Size distribution of the fragments was assessed with the BioAnalyzer High Sensitivity Chip (Agilent). We used the spreadsheet publicly available from Peterson et al. (2012) “Locus count from Bioanalyzer % in region” to calculate the number of fragments to be expected assuming a common genome size for sponges of ~300 Mb (Jeffery et al., 2013; Srivastava et al., 2010), and various size selections of RAD fragments. This number can subsequently be used to calculate the expected coverage when generating a known amount (Gb) of sequencing data. Custom‐made sample‐specific barcodes were ligated to the fragments to allow for the pooling of 21 samples per library, resulting in eight libraries in total. The Sage Science Pippin Prep was used to size‐select adapter‐ligated fragments of length 500–575 bp (indicating an insert size of 425–500 bp). A trial was run for 8, 10, and 12 polymerase chain reaction (PCR) cycles. In the end, 10 PCR cycles were chosen as a balance between DNA output and PCR duplication and were run on each library for enrichment and ligation of Illumina indices unique to each library pool. The quality and quantity of libraries throughout the process were checked using BioAnalyzer High Sensitivity chips (Agilent, Appendix S1 for an example). Libraries were pooled at equimolar volumes and 150 bp single‐end sequenced on Illumina HiSeq 2500 at the Vincent J. Coates Genomic Sequencing Facility at UC Berkeley.

### 2.3 Reference assembly, bioinformatic filtering, and genotype calling

Custom perl scripts were used for processing the resulting sequences (RADTOOLKIT v. 0.13.10, made available Supplemental Information). Raw fastq reads were demultiplexed using a maximum of one mismatch and removed if expected cut sites were not found. Resulting demultiplexed reads were trimmed of Illumina adapter contaminations and low‐quality reads using cutadapt v1.15 (Martin, 2011) and Trimmomatic (Bolger et al., 2014). Cleaned reads were clustered with CD‐HIT v4.6.1 (Fu et al., 2012; Li & Godzik, 2006), with a minimum support per cluster set at three reads, and representative sequences retained for each cluster. RepeatMasker v4.0 (http://repeatmasker.org/) was used to mask putative repetitive elements, low complexity regions, and short repeats using “Suberitidae” as a database (Smit et al., 2013). Loci were discarded if >60% of nucleotides per loci were Ns. The resulting RAD loci were combined for all individuals, and a de novo reference was built from loci shared by at least 70% of individuals.

We screened for loci from putative microbes in different ways. First, potential bacterial, viral, and human sequence contamination were removed via Blasting to reference sequences from GenBank following Maas et al. (2018) (see their Supplemental Table 1 for GenBank data used). Next, we ran Kraken v1 (Wood & Salzberg, 2014), a fast sequence classifier to BLAST (Altschul et al., 1990) our loci against bacterial databases with default settings. Third, we used BlobTools (Laetsch & Blaxter, 2017) to taxonomically partition reads and cut off loci with >55% GC content, as we expect sponge microbes to have higher GC content than sponge hosts (Horn et al., 2016). The identified microbial loci were filtered out using a custom‐made perl script (SNPcleaner, github.com/tplinderoth/ngsQC/tree/master/snpCleaner; Bi et al., 2013, 2019).

Cleaned sequence reads for each individual were aligned to the de novo generated reference separately using Novoalign v4.0 (http://www.novocraft.com), and only uniquely mapping reads were retained. Picard (www.picard.sourceforge.net) was used to add read groups, SAMtools v1.9 (Li et al., 2009) to generate a BAM file per individual, and GATK (McKenna et al., 2010) to perform realignment. SAMtools and BCFtools v1.2 were used to generate a VCF file. Single Nucleotide Polymorphisms (SNPs) and invariant sites were masked around 10 bp of an indel. Sites were removed if the depth was outside 1st and 99th percentile of the overall coverage. The custom perl script SNPcleaner was used for further filtering of SNPs. Ultimately, one random SNP per RADtag was retained for downstream analyses.

Calling SNPs and genotypes based on allele counts may be highly uncertain if coverage is low (Johnson & Slatkin, 2008; Lynch, 2008), which subsequently may bias downstream analyses. Therefore, we compared results from genotype calls and genotype likelihoods. Genotype likelihoods were generated via an empirical Bayesian framework via Analysis of Next‐Generation Sequencing Data (ANGSD v.0.930) (Korneliussen et al., 2014). We set genotype posterior probabilities of 0.95 as a threshold in ANGSD to output high‐confidence genotypes for analyses performed in GENODIVE v3.0 requiring genotype calls (Meirmans & Van Tienderen, 2004). For downstream analyses based on either genotype likelihoods and genotype calls, we tested the effect of coverage (3X and 10X) and missing data included (max. 30%, 10%, 5%, and 1% allowed missing data).

### 2.4 Detection of major lineages

We reconstructed phylogeographic relationships among and within lineages via a maximum likelihood tree. The maximum likelihood approach was performed via genotype calling and the software IQ‐Tree (Nguyen et al., 2015). First, we created consensus sequences in fasta format for all 125 individuals using ANGSD (Korneliussen et al., 2014), applying the options ‐doFasta 3 and ‐doCounts 1. Next, we concatenated the consensus sequences for all loci for each individual, resulting in a consensus sequence of 55 kb per individual, and carried out an alignment using MAFFT (Katoh & Standley, 2013). We then constructed the maximum likelihood phylogenetic tree using the IQ‐Tree software with 1000 ultrafast bootstraps and an SH‐like approximate likelihood test for 1000 replicates. The best fitting substitution model was inferred using the ‐m TEST function in IQ‐Tree.

Next, we explored admixture patterns using ngsAdmix (Skotte et al, 2013). Ancestry of populations was explored through calculating admixture proportions per individual and varying the estimated number of ancestral populations (*K*). The most likely *K* was determined by running 10 replicate runs of each respective *K*, calculating the log‐likelihood value of each, and choosing the value of the real *K* after which the likelihood plateaus or increases only slightly (Evanno et al., 2005). Differentiation among lineages was also assessed via an Analysis of Molecular Variance (AMOVA) with 1000 permutations using genotype calls (Excoffier et al., 1992). We used major lineages, two sublineages determined from the maximum likelihood tree, and populations as the nested levels. For the AMOVA, we used genotype calls allowing only 10% missing data to improve accuracy.

### 2.5 Population genomic structure and migration network of lineage B

We assessed population structure and differentiation within Lineage B using four approaches. We ran a Principal Component Analysis (PCA) based on a covariance matrix computed by ngsTools on genotype likelihoods (Fumagalli et al., 2014) and via GENODIVE using genotype calls (Meirmans & Van Tienderen, 2004). As an unsupervised clustering method, PCA estimates population genomic structure without bias. Next, we performed a neighbor‐joining network (NeighborNet) analysis using Splitstree using genotype likelihoods (Huson, 1998; Huson & Bryant, 2006). Splitstree does not force a tree‐like structure onto the data and thus can verify the extent to which the data conform to a hierarchical tree structure. Then, between‐population differentiation was assessed via normalized population differentiation was calculated using high‐confidence genotype calls in GENODIVE. Normalized fixation index (*F*'_ST_) was calculated to eliminate the effect of within‐population diversity (Meirmans & Hedrick, 2011). Finally, a migration network was constructed using Nei's G_ST_ with the threshold at 0.2 and 1000 bootstraps using the DiveRsity package in R (Keenan et al., 2013), as demonstrated by Sundqvist et al. (2016).

### 2.6 Genomic diversity and inference of demographic histories

We estimated the within‐population genomic diversity using two diversity measures. We calculated expected heterozygosity (H_e_) using GENODIVE, and overall heterozygosity and nucleotide diversity (π) using ANGSD (Nei, 1987). Population demographic histories were inferred via Stairway Plots using Site Frequency Spectra (SFS) (Liu & Fu, 2015). Stairway Plots offer an opportunity to infer demographic changes without requiring predefined models to test. Mutation rate is not known for sponges, but was calculated at 1.1 × 10^−8^ per generation via the regression coefficient by Lynch (2010) using the estimated genome size of 300 Mb. Generation time was set at 1 year. Stairway analyses were run for a subset of locations: two lagoon populations (Sea Australia and Sea Indonesia), two highly connected lakes (Papua4 and Papua30), and two isolated lakes (Papua1 and Papua5).

### 2.7 Associations to geographic distance, environmental variables, and degree of connection

Finally, we explored spatial, environmental, and oceanographic associations to genomic structure. Mantel tests (Legendre & Legendre, 2012; Mantel, 1967) were used to test significance of correlations between genomic, geographic, environmental, and connection distance matrices. For genomic distances, we used normalized pairwise genomic differentiation (*F*'_ST_/(1−*F*'_ST_)). Geographic distance was calculated as minimum pairwise distances in meters between lakes using lake coordinates as input for the *geosphere* package in R. Using averages of temperature (°C) and salinity (ppt) per lake, we performed a Principal Component Analysis (PCA) to calculate informative scores. The scores from all retained PCA axes were then used computing the environmental distance matrix using the function *dist* in R. Here, low distance values indicate lakes with a similar environment in terms of temperature and salinity, whereas high distance values indicate lakes with very dissimilar environments. Connection distance was calculated following the equations of Maas et al. (2018), so that a high distance value indicated a pairwise comparison of isolated lakes and a low distance value indicated a pairwise comparison of connected lakes, with all other comparisons falling in between, so that the resulting matrix would reflect resistance values. Mantel tests were run with 10,000 permutations using *vegan* in R. We verified the absence of autocorrelation between geographic, environmental and connection distances using Mantel's tests. Finally, we performed Spearman correlation tests among genomic diversity indices per lake with temperature, salinity, degree of connection and lake area. Correlations of *r* ≥ .5 were considered strong, and alpha was set to 0.01.

## 3 RESULTS

### 3.1 Lake characterization

The physical and environmental profiles of the two lagoons and nine marine lakes are provided in Table 1. In general, we observed higher temperatures (30.8°C ± 1.2°C) and lower salinities (27.3 ppt ± 2.7 ppt) in lakes when compared to the open lagoon locations (29°C and 33.5 ppt). Connection to the surrounding sea varied among lakes, with highly connected to highly isolated lakes based on tidal amplitudes. For instance, lake Papua4 was found to have the highest connection with tidal amplitude representing 80% of that of the surrounding sea, indicating high water exchange with the sea, while lake Papua1 was most isolated, with the tidal amplitude only being <10% of the surrounding sea, and indicating limited exchange with the sea. To illustrate, the highly connected lakes had large enough channels or caves for fish to swim through, and we could see water flooding in during high tides. We categorized two lakes with high connection to the sea, three lakes with medium connection to the sea, and four isolated lakes with low connection to the adjacent sea (Table 1).

### 3.2 Read statistics and filtering

After sequencing and demultiplexing, we obtained 1,127,497,643 reads from 168 sponges. On average, we obtained 7,673,269 reads per individual. Individuals with less than 2,000,000 reads were removed from subsequent analyses. Based on the calculation table from Peterson et al. (2012), an estimated genome size of 300 Mb, and a size selection of 425–500 bp, we expected to retain 13,652 RADtags. This was close to the actual retained loci, as the de novo reference retained 14,442 tags when keeping RADtags with at least 3× coverage and present in at least 70% of individuals. Kraken and Blobtools identified 15 out of the 14,442 RADtags containing possible bacterial contamination, which mapped to *Synechococcus* sp., a Cyanobacteria genus. These were removed from the data set (tags available in Supplemental Information).

After filtering, we retained 125 sponges with 973,697,804 reads in total, with coverage ranging from 3.1–82.2× (average 24.0×). The number of genomic markers retained strongly varied depending on the inclusion or exclusion of the two major lineages. When including both lineages 541 SNPs were retained, yet when only including Lineage B the number of SNPs increased almost ninefold to 4826. For Lineage B, in total 23,742 SNPs were called over all tags, but after selecting one SNP per tag we retained the 4826 SNPs for subsequent analyses. Also for Lineage B, depending on the filtering options (genotype calls or genotype likelihoods, coverage 3× or 10×, included missing data 30%, 10%, 5% or 1%) the number of SNPs varied from 56 to 4826 (Table S2).

### 3.3 Detection of major lineages and sub‐lineages

**FIGURE 2:**
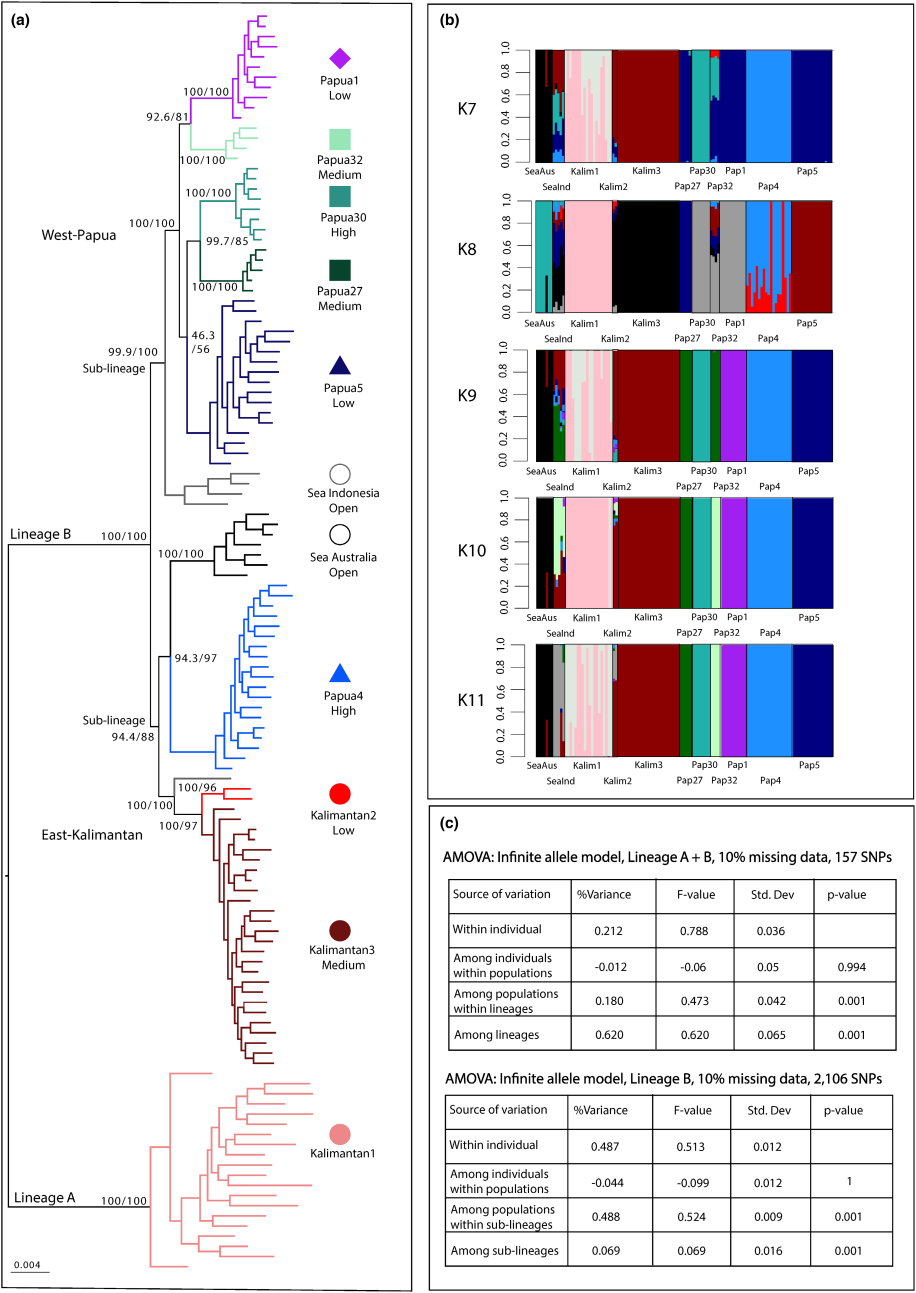
Distinction of major genetic lineages A and B of sponge populations. (a) Maximum Likelihood IQTree using consensus sequences of 125 individuals based on 541 SNPs. Bootstrap values for major branches are displayed. (b) Bayesian admixture analysis for putative ancestral populations (*K* 7–11) based on genotype likelihoods via ngsAdmix. Each bar represents one individual. *K* = 9 was indicated as the most likely ancestral populations. (c) Analysis of Molecular Variance (AMOVA) for Lineage A and B (top), and only Lineage B (bottom). Amount of variance explained in percentage, *F*‐values and significance values are displayed. Colors and codes correspond to Figure 1 and Table 1.

A maximum likelihood phylogenetic tree based on 541 SNPs showed two divergent lineages for 125 individuals (Figure 2a). These lineages are concordant with Lineage A and B as defined by Becking et al. (2013). Lineage A was only represented by individuals of Kalimantan1. The remaining populations in marine lakes fell under Lineage B. Within Lineage B, two sublineages could be seen representing on the one hand locations from East‐Kalimantan, Australia and Papua4, and on the other the rest of the locations from West‐Papua. Lagoon population Sea Indonesia was a sister group to both the East‐Kalimantan and West‐Papua sub‐lineages.

Admixture analyses showed Kalimantan1 to be consistently distinct from all other populations throughout the range of ancestral populations *K* (Figure 2b). The most likely number of ancestral populations was found to be at *K* = 9 (Figure S1), after which new groups did not add additional information. At *K* = 9, all marine lakes are uniquely colored, except Papua27 and Papua32, which are grouped together (dark green), and Kalimantan2, which showed admixture from multiple groups. Furthermore, Sea Indonesia and Sea Australia also showed admixture from multiple groups.

When analyzing the complete dataset, the AMOVA showed most variation to be explained by the two major lineages (A and B) (Figure 2C, 62%). Within Lineage B, most variation was explained by differences among populations (48.8%). Variation among the two sublineages (East‐Kalimantan, Australia and Papua4 on the one hand and the other populations of West‐Papua on the other) was also found to significantly contribute to the variation, but only 7%. All subsequent analyses were run for Lineage B for 105 sponge individuals with 56–4826 SNPs, depending on the filtering option.

### 3.4 Population genomic structure and migration network of lineage B

We tested different filters to assess the influence on the genomic patterns. Within Lineage B, patterns remained highly similar for all filters (see Figures S2–S4 and Tables S3–S6). Therefore, all further reported analyses were performed filtering on 3× coverage and max. 30% missing data, as this retained the most SNPs and thus would result in the highest resolution.

**FIGURE 3:**
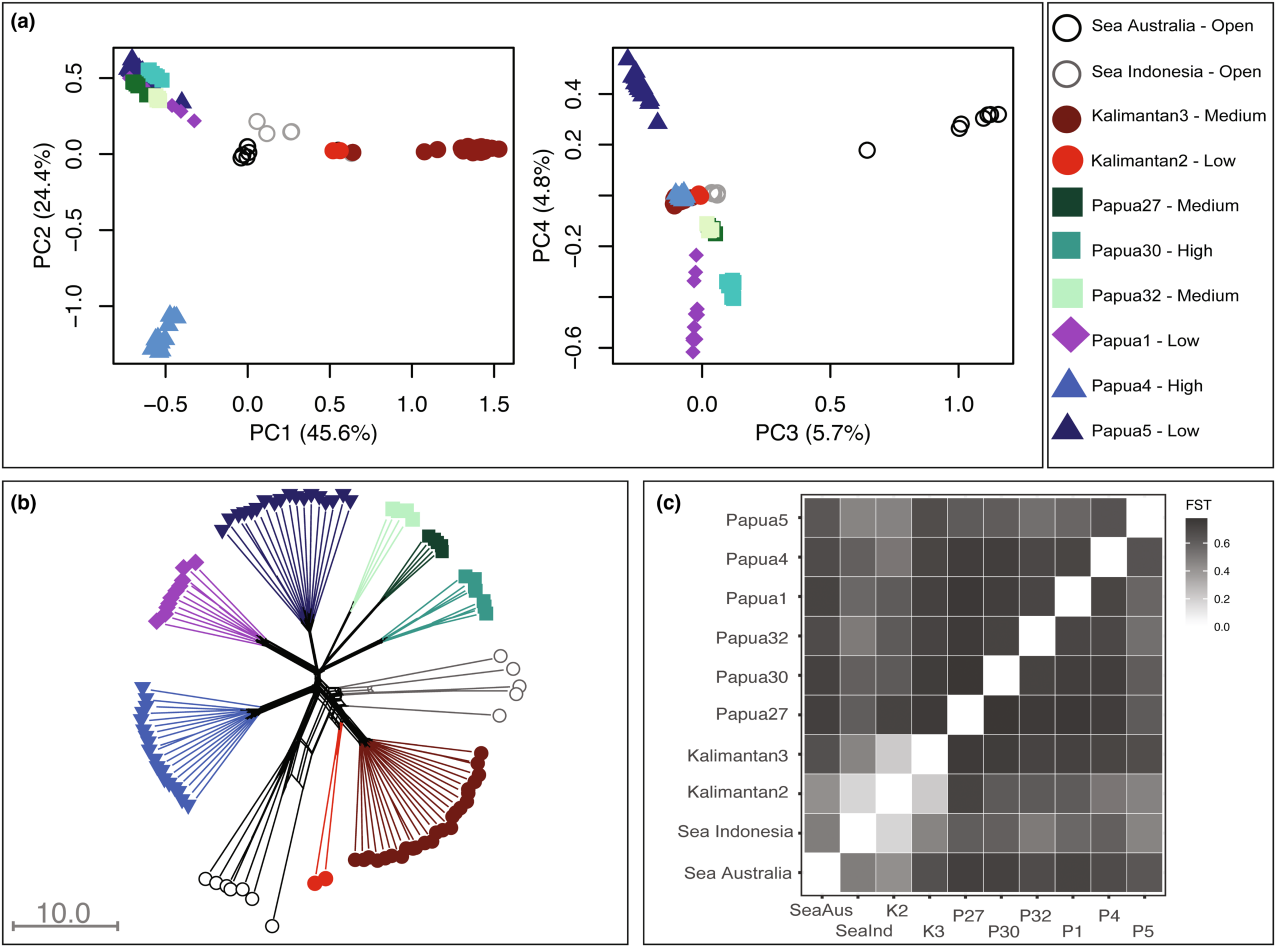
Population genomic structure analyses for Lineage B from sponge populations. (a) Principal Component Analysis (PCA) based on pairwise covariance. Each dot represents one individual. (b) Neighbor‐Joining Network with equal angles computed in Splitstree based on pairwise genomic distances. (c) Heatmap of normalized *F*'_ST_ (values in Table S5). Colors and codes correspond to Figure 1 and Table 1.

The sponge individuals clustered by lake and lagoon location in the Principal Component Analysis (PCA) (Figure 3a, Figures S2 and S3). The first four Principal Components (PCs) explained 80.5% of total variation (Figure 3a). PC1 explained 45.6% of the variation, separating populations by geographic region, with the lakes from West‐Papua being distinct from the lakes in East‐Kalimantan. PC2 explained 24.4% of variation, separating lake Papua4 from the other lakes. The lagoon populations (Sea Australia and Sea Indonesia) clustered towards the center of the graph. PC3 and PC4 (explaining 10.5% in total) further separated Sea Australia and lakes Papua5, and to a lesser extent Papua1 and Papua30. Lakes Papua27 and Papua32 remained closely associated in both plots. High and medium connected lakes did not cluster together, nor with the open sea populations.

Findings from the Splitstree network were consistent with patterns found for the PCA (Figure 3b, Figure S4). The network showed a high fit (fit = 99.2) and a small degree of reticulation (*d* = 0.153), indicating a tree‐like structure. The sea populations Sea Australia and Sea Indonesia showed higher reticulation than the marine lake populations, indicating potential introgression or hybridization events. All marine lake populations were diverged and showed no reticulation.

Pairwise fixation indices (*F*'_ST_) showed high levels of genomic structuring (0.63 ± 0.13) (Figure 3C). When including Lineage A, the comparisons of populations to Kalimantan1 showed *F*'_ST_ values of >0.8 (Figure S5). Within Lineage B, the *F*'_ST_ values ranged from 0.18 between Sea Indonesia and Kalimantan2, to 0.78 between Papua30 and Papua32 (Table S3). All pairwise comparisons were significant, except for the comparison between Papua32 and Kalimantan2, likely due to sample size (*n* = 4 and 2, respectively). Striking was that the *F*'_ST_ values among populations from highly connected lakes were equally strong as among highly and low connected lakes.

The migration network among lakes indicated the strongest relative bidirectional migration between marine lakes and the lagoon population in East‐Kalimantan (Figure 1D). Lagoon population Sea Indonesia was linked to some degree to all other populations (relative bidirectional migration 0.2–1). Within West‐Papua, bidirectional migration was observed between Papua5 and four other lakes (Papua27, Papua30, Papua32, and Papua1). Lagoon population Sea Australia showed links to Kalimantan2 and 3, and Papua5. There was no apparent strong bidirectional migration among the high and medium connected lakes, nor among those and the sea.

### 3.5 Genomic diversity and demographic inferences per population

Population genomic diversity varied among lakes (Table 1, Table S4). The highest genomic diversity was consistently found for the lagoon populations Sea Australia and Sea Indonesia, as seen for nucleotide diversity (π) (0.0101 and 0.0095, respectively), and for the expected heterozygosity (H_e_) (0.157 and 0.117, respectively). Low genomic diversity was consistently observed in populations Papua1 (π = 0.0036, H_e_ = 0.054) and Papua27 (π = 0.0037, H_e_ = 0.038). However, for populations Kalimantan2 and Papua5, there were differences in estimates on diversity depending on which method was chosen. When estimating heterozygosity from genotype likelihoods via ANGSD, we found high diversity for Kalimantan2 (0.1200) and low diversity for Papua5 (0.0188), yet when estimating expected heterozygosity via GENODIVE we found low diversity for Kalimantan2 (0.034) and high diversity for Papua5 (0.095). For Kalimantan2, this may be an artifact of low sample size (*n* = 2), as GENODIVE takes sample size into account. These were the only two lakes in which such a remarkable difference between estimates was observed.

**FIGURE 4:**
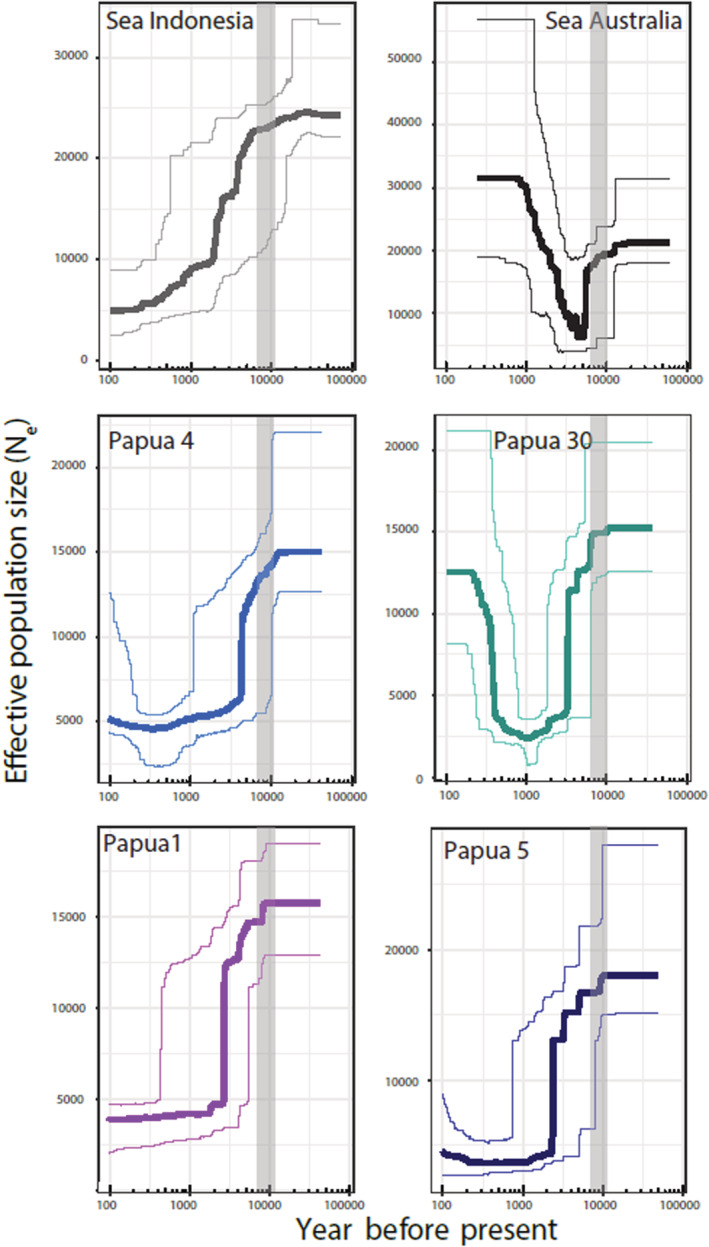
Demographic history inferences on sponge populations within Lineage B. Demographic histories of six locations are displayed: lagoon populations Sea Indonesia and Sea Australia (top), highly connected marine lakes Papua 4 and Papua30 (middle), and isolated lakes Papua1 and Papua5 (bottom). Mean (dark) and 12.5/87.5% confidence intervals are displayed. The gray box indicates putative filling of marine lakes after the Last Glacial Maximum (8000–10,000 years ago).

We assessed changes in effective population size for six locations using Stairway plots (removing populations with small sample sizes from the analysis; Figure 4). All locations showed a decrease in effective population size after the Last Glacial Maximum (approximately 20,000 years ago). For the lagoon population Sea Australia and the highly connected lakes Papua27 and Papua4, this bottleneck was followed by an expansion (although less pronounced for Papua4). For lagoon population Sea Indonesia and low connected lakes Papua1 and Papua5 no notable subsequent expansion was observed.

### 3.6 Association to geographic distance, environmental variables, and degree of connection

Spearman correlation tests indicated that within‐population genomic diversity (nucleotide diversity π and heterozygosity H_e_) was not influenced by lake area (π: Spearman's rho = 0.03, *p* = .95, H_e_: rho = −0.06, *p* = .88), degree of connection (π: rho = 0.43, *p* = .25, H_e_: rho = 0.53, *p* = .15), salinity (π: rho = 0.37, *p* = .33, H_e_: rho = 0.24, *p* = .53), or temperature (π: rho = −0.61, *p* = .08, H_e_: rho = −0.20, *p* = .60) (Table S5, Figure S6).

Mantel tests, furthermore, indicated no correlation between the geographic and genomic distance matrices (*r* = 0.007, *p* = .50) (Figure S7A). This was consistent when repeating the analysis for all filter options (Table S6). Finding no correlation refutes the isolation‐by‐distance hypothesis and indicates other factors might explain the distribution of *S. diversicolor* genomic diversity. However, the genomic distance matrix also did not correlate with matrices of environmental distance (*r* = 0.002, *p* = .503, Figure S7B), or connection distance (*r* = 0.041, *p* = .441, Figure S7C). Therefore, we did not find evidence for isolation‐by‐environment or isolation‐by‐resistance patterns.

## 4 DISCUSSION

The existence of multiple independently derived populations in marine lakes provides an opportunity for fundamental research into the role of isolation in population divergence in marine taxa. By comparing sponge populations in the Indo‐Pacific from marine lakes and lagoons at different spatial scales, environmental conditions, and degree of connection to the sea, we detected high levels of genomic differentiation across the studied geographic area and provided new evidence of small scale structure for sessile species with a short dispersive larval stage. The structure of the populations could, however, not be explained by geographic distance, local environments or degree of connection to the sea alone. Our work exemplifies that a higher resolution of markers retained with reduced representation genome sequencing can elucidate small‐scale population genomic structure which are not visible with single marker studies. In general, we showed that old extractions yielding low‐coverage data from nonmodel organisms can readily be used in population genetic and genomic analyses to study small‐scale population differentiation. Below, we discuss our findings and potential implications for future phylogeographic and population genomic studies on sponges.

### 4.1 RADseq reveals small scale structure in Suberites diversicolor

The sponge populations show strong population structure and high levels of genomic differentiation across the study area. These results are in concordance with our hypothesis based on the life history traits of benthic sponges with a predominantly sessile life cycle and short larval durations (Maldonado, 2006). Restriction site‐associated DNA sequencing proved suitable to retrieve major genetic lineages in the sponge *S. diversicolor*, which had been split in Lineage A and B by Becking et al. (2013), using the Cytochrome Oxydase I (*COI*) and the Internal Transcribed Spacer 1&2 (*ITS*) nuclear markers. Investigation of morphological traits based on spicules and analyses of *COI* and *ITS* markers determined that Lineage B is likely one species, yet did not show any spatial intraspecific variation across populations (Becking et al., 2013). Diving deeper into Lineage B, the thousands of SNPs that the RADseq approach of the current study provided revealed small‐scale (<10 km) genomic patterns for *S. diversicolor* that had not previously been shown. We observed high intralineage genomic differentiation (*F*_ST_ range 0.18–0.78), with admixture PCA and Splitstree analyses showing clear clustering by marine lake.

Finding more structure when using higher numbers of genomic markers has previously been shown for other marine organisms (e.g., D'Aloia et al., 2020; Lemopoulos et al., 2019; Maas et al., 2018; Sunde et al., 2020; Timm, 2020). Thus, increasing the marker panel can result in the reassessment of previously assumed panmictic populations. Particularly in the highly diverse Indo‐Pacific, population genomic studies are increasingly revealing high population structure (Hernawan et al., 2017; Lal et al., 2017; Vu et al., 2020). The signature of strong population differentiation observed for the marine lake sponges using high‐resolution data now better matches those of co‐distributed species in marine lakes (Dawson & Hamner, 2005; de Leeuw et al., 2020; Maas et al., 2018). Population genomic studies point toward the role of both historical and contemporary processes in establishing the current population genomic structure. Yet for marine invertebrates, especially sponges, the underlying mechanisms driving the structure are poorly understood (Oleksiak & Rajora, 2020; Pérez‐Portela & Riesgo, 2020).

### 4.2 Influence of geographic distance, environment and connection on genomic structure and diversity

The results highlighted the effects of several drivers of sponge population diversity and structure. First, the analysis of genomic differentiation showed that population similarity among sponges did not decay with increasing geographic distance. While populations were strongly clustered per marine lake, with a clear regional split between populations from East‐Kalimantan and West‐Papua, we did not observe a pattern of isolation‐by‐distance. This is remarkable, as we sampled at a wide range of geographical distances (1–1400 km). Previous studies using a low number of markers did find a pattern of isolation‐by‐distance for sponges in oceanic locations (Blanquer & Uriz, 2010; Duran et al., 2004; Noyer & Becerro, 2012; Pérez‐Portela et al., 2014). Therefore, we would have expected the ocean populations seeding the lake populations to show isolation‐by‐distance patterns, which would then be reflected in the lakes even if they had no ongoing gene flow among the individual lakes. However, as we could only sampled two lagoon populations, it is possible that we did not sample the actual seeding populations. Secondly, we also did not detect a pattern of isolation‐by‐environment (Nosil et al., 2009; Orsini et al., 2013; Rundle & Nosil, 2005), despite the great environmental disparity among lakes (temperature: 29–32.4°C, salinity: 24–33.4 ppt). This finding contrasts another study on sponges reporting an influence of environmental heterogeneity (temperature and productivity; Giles et al., 2015) on population structure. As there is clearly a “lake‐effect,” we may not have measured the environmental variables that are relevant for the structuring. Finally, we did not find evidence for higher gene flow among the highly connected lakes as compared to isolated lakes, disputing the presence of an isolation‐by‐resistance pattern. Other studies did find evidence for dispersal limitations through oceanographic currents (Chaves‐Fonnegra et al., 2015; Richards et al., 2016; Riesgo et al., 2019).

Our results indicate that mechanisms other than dispersal limitation or divergence due to differences in local environments are important in structuring *S. diversicolor* populations. Perhaps *S. diversicolor* populations are isolated in the lake environment, irrespective of the degree of connection of the lake to the adjacent sea, where the low dispersal ability of sponges restricts effective gene flow. Populations can then become differentiated through genetic drift or via local adaptation to environmental parameters not yet recorded (Frankham et al., 2002). The observation of a severe population bottlenecks with subsequent recovery in Papua30, and population declines with little‐to‐no expansion in Papua1, Papua4 and Papua5, suggests a potential effect of founder events, which in turn leads to increased drift effects. Founder effects and subsequent priority effects could explain the pattern of strong population structure (De Meester et al., 2016; Fukami, 2015; Orsini et al., 2013).

Priority effects were previously discussed as potential drivers of structure in marine lake organisms by Maas et al. (2018) and de Leeuw et al. (2020). Depending on the spatial scale that was analyzed, Maas et al. (2018) found an effect of geographic distance and connectivity influencing mussel population structure. They argued that while founder events can stochastically drive alleles to fixation in small populations, ongoing dispersal would overwhelm this effect (Mayr, 1963; Waters et al., 2013). Mussels have extensive pelagic larval duration periods, and Maas et al. (2018) hence argued that priority effects mediated by local adaptation could facilitate the observed patterns of population structure (De Meester et al., 2016; Fukami, 2015; Orsini et al., 2013). Sponges, in contrast, generally have poor dispersal abilities (Maldonado, 2006). As the current study does not find an effect of connection to the sea in structuring populations, stochastic fixation of alleles due to genetic drift amplified by founder events may more likely be the cause of each population being distinct. Future studies should further attempt to decouple genetic drift from migration, for instance by using a rigorous modeling approach as provided by fastsimcoal (Excoffier & Foll, 2011), or equivalent.

### 4.3 Implications for sponge phylogeography and population genomic studies

The RADseq strategy effectively detected two major genetic lineages (Lineage A and B) (similar to Becking et al., 2013). When combining both lineages, significantly fewer markers were recovered than when analyzing the lineages separately. Based on our filtering strategy, we retained 541 SNPs when including both lineages, compared with 4826 SNPs when analyzing only Lineage B. This reflects a more than 90% loss of common markers and indicates that the resolution of RADseq generated markers can be less effective when multiple lineages are (unknowingly) included. Given that morphologically cryptic species are prevalent in sponges (e.g., Becking, 2013; Morrow & Cárdenas, 2015; Swierts et al., 2013), it may be advised to first verify broad genetic lineages using traditional single markers before starting an extensive sponge population genomic study implementing high‐resolution markers.

While traditionally single markers have been successful in identifying sponge species complexes, they have often lacked the resolution for studies on intraspecific population genetic diversity (as reviewed in Pérez‐Portela & Riesgo, 2020; Uriz & Turon, 2012; Van Oppen et al., 2002; Wörheide et al., 2005), with notable exceptions (Klautau et al., 1999; Wörheide et al., 2002, 2008). Using the high resolution provided by RADseq generated markers allowed us to detect clear clustering by lake even on very small spatial scales: 1–10 km. The scale at which we find strong structure is smaller compared with recent studies using microsatellites in the sponges *Xestospongia muta* (Richards et al., 2016), *Paraleucilla magna* (Guardiola et al., 2016), *Plenaster cragi* (Taboada et al., 2018), and *Petrosia ficiformis* (Riesgo et al., 2019). Previous studies using SNPs at higher resolution also revealed little structure at small spatial scales, with Brown et al. (2017) detecting little structuring for *Aphrocallistes vastus* in British Colombia at scales of <275 km and Leiva et al. (2019) finding panmixia at scales <900 km for *Dendrilla antarctica*. It could be that these are indeed highly connected populations, possibly through rafting or sperm‐mediated gene flow (DeBiasse et al., 2014; Maldonado, 2006). Yet, it is also possible that the number of SNPs from Brown et al. (2017) and Leiva et al. (2019) (67 and 529, respectively) was too low to detect subtle structure at small scales. However, even when using a larger SNP dataset, Taboada et al. (2022) found high connectivity on a geographic range of 2500 km for the deep‐sea sponge *Phakellia ventilabrum*, suggesting findings of connectivity can be ecosystem‐ and species‐specific.

A challenge in high‐resolution genomic studies is presented by potential effects on downstream analyses by different bioinformatic filtering strategies. We rigorously tested the effects of several bioinformatic genotype calling and filtering procedures one can opt for when working with RADseq data. We tested the effect of coverage (3× or 10×) and missing data included (max. 30%, 10%, 5% or 1%). Additionally, we tested the effects of using either genotype calls used by GENODIVE (Meirmans & Van Tienderen, 2004), or genotype likelihoods used by ANGSD (Korneliussen et al., 2014). While the stringency of the filter had severe effects on the number of SNPs included in the analyses (4826 in the most relaxed option versus 56 in the strictest option, Table S2), the downstream analyses showed consistent results over all filtering options (Figures S2–S4, Tables S3–S5). The patterns of highest versus lowest genomic nucleotide diversity, heterozygosity and expected heterozygosity (Table S3), highest versus lowest genetic differentiation (*F*'_ST_, Table S4), and associations of diversity with environmental predictors (Table S5) remained remarkably consistent. Hence, when patterns are as strong as we find for the marine lakes in the Indo‐Pacific, it matters little how many SNPs are used or how strictly coverage or amount of missing data is selected for. Although when using very few SNPs, the population structure observed in the PCAs did begin to dissociate (Figures S2 and S3). Other studies using RADseq data found evidence for both a high effect (Marandel et al., 2020; Shafer et al., 2017) and a low effect (Tripp et al., 2017) of filtering on downstream analyses. In general, we recommend each study to perform a sensitivity analysis regarding filtering options and downstream results, as it may be different for each specific study system (Díaz‐Arce & Rodríguez‐Ezpeleta, 2019).

Another challenge to unveiling sponge population genomic patterns is the association between the host sponge and its microbial communities (Webster & Thomas, 2016). Since sponges have the propensity to harbor dense communities of microbes, there is a potential of host genomic material being contaminated by that of microbes, thus clouding host‐specific patterns. As microbial biodiversity patterns can differ from sponge host diversity (Noyer & Becerro, 2012), it is important to filter these out. We screened for loci from putative microbes using different filtering strategies and feel confident that the results reflect host diversity. Furthermore, the lack of congruence between the host population structure and previously published microbial communities in *S. diversicolor* from the same locations (Cleary et al., 2013; Ferreira et al., 2020) confirmed that the rigorous bioinformatic filtering removed associated microbes. However, microbial contamination remains a point of attention for future studies on sponge population genomics using RADseq.

Finally, our adjustments to the existing low‐cost protocol of Peterson et al. (2012) with a step‐by‐step protocol presented in the supplement can help retrieve extensive data for non‐model marine organisms in general and tropical sponges in particular, thus benefitting future studies. We further showed that reduced representation genome sequencing can work for DNA extracted for other purposes and stored for long times in a −20°C freezer before sequencing, or suboptimal removal of contamination before sequencing. Recent developments with capture‐based methods such as hyRAD (Suchan et al., 2016) can further exploit the potential of older DNA extractions. This gives hope to the wealth of knowledge to be gained from extractions from past studies across the world.

## 5 CONCLUDING REMARKS

In conclusion, we observed high population genomic structure for the sponge *S. diversicolor* even on small spatial scales of <10 km that was previously undetected using single markers. This finding reflects the life history of the sponge with short larval durations, but also highlights the importance of higher genomic resolution to detect structure. Based on this dataset, we could not link the observed strong genomic differentiation to any of the expected predictors (geographic distance, local environments or extent of connection to the sea). As we observed population bottlenecks for all marine lake locations, regardless of their extent of connection to the sea, other factors such as strong founder events coupled with priority effects could be at play. A major objective of marine molecular ecology is to obtain accurate estimates of genomic structure, as it can inform efforts to identify units of management and design effective marine protected areas (MPAs) (Kelley et al., 2016; Selkoe et al., 2016). Our results call for a reassessment of population connectivity of poorly dispersing organisms such as sessile benthic organisms, as previous assumptions of panmixia may be an artifact of low‐resolution markers. In turn, these studies can then more accurately inform the determination of relevant spatial scales of MPA networks.

## AUTHOR CONTRIBUTIONS

**Diede L. Maas:** Formal analysis (lead); investigation (equal); methodology (lead); visualization (lead); writing – original draft (lead); writing – review and editing (lead). **Stefan Prost:** Formal analysis (supporting); methodology (supporting); software (equal); supervision (supporting); visualization (supporting); writing – review and editing (supporting). **Christiaan A. de Leeuw:** Data curation (supporting); investigation (supporting); methodology (supporting); writing – review and editing (supporting). **Ke Bi:** Formal analysis (supporting); methodology (supporting); resources (equal); software (lead); writing – review and editing (supporting). **Lydia L. Smith:** Formal analysis (supporting); methodology (supporting); supervision (supporting); writing – review and editing (supporting). **Purwanto Purwanto:** Resources (supporting); writing – review and editing (supporting). **Ludi P. Aji:** Resources (supporting); writing – review and editing (supporting). **Ricardo F. Tapilatu:** Resources (supporting); writing – review and editing (supporting). **Rosemary G. Gillespie:** Supervision (supporting); writing – review and editing (supporting). **Leontine E. Becking:** Conceptualization (lead); data curation (lead); formal analysis (supporting); funding acquisition (lead); investigation (lead); methodology (lead); supervision (lead); visualization (supporting); writing – original draft (equal); writing – review and editing (equal).

## ACKNOWLEDGEMENTS

The following people provided help with logistics during fieldwork and/or labwork: . B. Alvarez, M. Ammer, Bahruddin, H. Breeuwer, L. Dong, M. Erdmann, B. Hoeksema, P. Kuperus, S. C. Lim, S. Menken, A. Miners, A. Oherenan, K. Peijnenburg, W. Renema, Suharsono, R. Tapilatu, Y. Tuti, B. Voetdijk, N. J. de Voogd, the staff of Nabucco Island Dive Resort, Derawan Dive Resort, Misool Eco resort, Papua Diving. We are grateful for useful discussions with Dr. P. Meirmans. This work used the Vincent J. Coates Genomics Sequencing Laboratory at UC Berkeley, supported by NIH S10 Instrumentation grants S10RR029668 and S10RR027303. The Netherlands Organisation for Scientific Research provided funding to Leontine E. Becking through the grants RUBICON #825.12.007, VENI #863.14.020 and VI.Vidi.193.137. Fieldwork in Indonesia was made possible through additional financial support of National Geographic Society Waitt Grant, De Beukelaar‐van der Hucht Stichting, World Wildlife Fund, Netherlands‐INNO Fund, the Schure‐Beijerinck‐Popping Fund of the Royal Dutch Academy of Science (KNAW), Conservation International Ecosystem Based Management program (funded by the David and Lucile Packard Foundation), the Treub‐ Maatschappij Fund, the Leiden University Fund (LUF)/Slingelands, Singapore Airlines, the A.M. Buitendijk Fund and the J. J. ter Pelkwijk Fund (Naturalis Biodiveristy Center). The funders had no role in the study design, data collection and analysis, decision to publish, or preparation of the manuscript. We are grateful to the Indonesian Institute of Sciences (LIPI) and the Indonesian Ministry of Research and Technology (RISTEK) for providing the research permits (0094/FRP/SM/V/2009;098/SIP/ FRP/SM/V/2011;098/SIP/FRP/SM/V/20113246/FRP/SM/VII/2012;#3B/TKPIPA/E5/Dit.KI/III/2016).

## FUNDING INFORMATION

Nederlandse Organisatie voor Wetenschappelijk Onderzoek (Rubicon #825.12.007; VENI #863.14.020; VI.Vidi.193.137), NIH S10 Instrumentation grants (S10RR027303; S10RR029668), Leiden University Fund, National Geographic Society Waitt Grant, De Beukelaar‐van der Hucht Stichting, A.M. Buitendijk Fund, J.J. ter Pelkwijk Fund, WWF, Treub‐Maatschappij Fund, Singapore Airlines, Netherlands‐INNO Fund, David and Lucile Packard Foundation, Schure‐Beijerinck‐Popping Fund.

## CONFLICT OF INTEREST STATEMENT

The authors declare no conflict of interest.

## PERMISSION TO REPRODUCE MATERIALS FROM OTHER SOURCES

None.

## Supporting information


Figures S1–S7
Tables S1–S7

Appendix S1

Appendix S2

## Data Availability

Data and customary scripts are accessible via the Dryad Digital Repository (https://doi.org/10.5061/dryad.5mkkwh79w). Data consists of aligned reads in bam format for all 125 individuals sequenced, with an accompanying text file assigning the individuals and the de‐novo reference file in fasta format. Additionally, the tags mapped to microbes are included. Finally, custom scripts RADTOOLKIT v. 0.13.10 and SNPcleaner224.pl are stored here.
