# Supplementary material for: Sponge diversification in marine lakes: Implications for phylogeography and population genomic studies on sponges
Source: Ecol Evol. 2023 Apr 13;13(4):e9945. doi: 10.1002/ece3.9945 (PMC10099488; doi:10.1002/ece3.9945)
Supplement: Supplementary file 1 — Figures S1–S7 Tables S1–S7 [file ECE3-13-e9945-s003.pdf]

Supplemental Information for:

Sponge diversification in marine lakes: implications for phylogeography and population genomic studies on sponges

D.L. Maas, S. Prost, C.A. de Leeuw, K. Bi, L. Smith, Purwanto, L.P. Aji, R.F. Tapilatu, R. Gillespie, L.E. Becking

Table of contents

ST1: Comparison location codes	Page 2
ST2: Retained SNPs for filters	Page 2
ST4: Normalized F'_{ST} values	Page 3
ST5: Genetic diversity indices	Page 4
ST6: Diversity indices correlations	Page 5
ST7: Mantel test results	Page 5
SF1: Admixture analyses boxplots	Page 6
SF2: PCAs on genotype likelihoods	Page 7
SF3: PCAs on genotype calls	Page 8
SF4: Splitstrees	Page 9
SF5: Heatmap of F'_{ST}	Page 10
SF6: Diversity indices correlations	Page 10
SF7: Mantel tests	Page 11

Supplemental Table 1: Comparison of location codes used in the current study and overlapping locations of Becking *et al.* (2013).

Code<br>Current study	Code<br>Becking *et al.* (2013)
Sea Australia	DAR
Sea Indonesia	BER
Kalimantan1	KKB
Kalimantan2	TBB
Kalimantan3	HBL
Papua27	
Papua30	CAS
Papua32	URA
Papua1	RAJ
Papua4	MIS
Papua5	

Supplemental Table 2: Retained Single Nucleotide Polymorphisms (SNPs) for genotype likelihoods and genotype calls after various filtering options. Coverage was set at 3X or 10X, missing data allowed was 30%, 10%, 5% or 1%.

Filtering	SNPs<br>Genotype<br>likelihoods	SNPs<br>Genotype<br>calls
Cov3_Miss30	4,826	4,790
Cov3_Miss10	1,716	2,106
Cov3_Miss5	837	1,483
Cov3_Miss1	276	951
Cov10_Miss30	1,295	1,286
Cov10_Miss10	292	893
Cov10_Miss5	130	612
Cov10_Miss1	56	368

Supplemental Table 4: Normalized F'_{ST} and associated significance based on different filtering options for *Suberites diversicolor* populations. Below diagonal normalized F'_{ST} is displayed, above diagonal p-values. Tables are colored according to F'_{ST} values from low (green) to high (red).

**u3k70**	Sea Australia	Sea Indonesi	Kalimantan3	Kalimantan2	Papua30	Papua27	Papua32	Papua1	Papua4	Papua5
Sea Australia		0.002	0.001	0.034	0.001	0.001	0.007	0.001	0.001	0.001
Sea Indonesia	0.495		0.001	*0.051*	0.001	0.014	0.01	0.002	0.001	0.001
Kalimantan3	0.661	0.464		0.001	0.001	0.001	0.001	0.001	0.001	0.001
Kalimantan2	0.431	0.182	0.217		0.024	0.048	*0.067*	0.01	0.003	0.003
Papua30	0.727	0.598	0.751	0.619		0.002	0.003	0.001	0.001	0.001
Papua27	0.736	0.609	0.761	0.723	0.778		0.01	0.001	0.001	0.001
Papua32	0.684	0.509	0.718	0.622	0.723	0.76		0.002	0.001	0.001
Papua1	0.724	0.573	0.727	0.616	0.74	0.771	0.71		0.001	0.001
Papua4	0.682	0.601	0.716	0.521	0.741	0.746	0.699	0.716		0.001
Papua5	0.64	0.466	0.672	0.469	0.614	0.615	0.541	0.578	0.653	

**u10k70**	Sea Australia	Sea Indonesi	Kalimantan3	Kalimantan2	Papua30	Papua27	Papua32	Papua1	Papua4	Papua5
Sea Australia		0.001	0.001	0.035	0.001	0.002	0.003	0.001	0.001	0.001
Sea Indonesia	0.494		0.001	0.048	0.003	0.012	0.007	0.001	0.001	0.001
Kalimantan3	0.654	0.433		0.005	0.001	0.001	0.001	0.001	0.001	0.001
Kalimantan2	0.506	0.211	0.183		0.021	0.045	*0.074*	0.01	0.004	0.003
Papua30	0.725	0.611	0.738	0.718		0.001	0.004	0.001	0.001	0.001
Papua27	0.724	0.618	0.749	0.762	0.785		0.007	0.002	0.001	0.002
Papua32	0.688	0.548	0.716	0.674	0.727	0.772		0.001	0.001	0.001
Papua1	0.708	0.585	0.72	0.641	0.713	0.765	0.717		0.001	0.001
Papua4	0.682	0.596	0.701	0.597	0.747	0.746	0.706	0.721		0.001
Papua5	0.646	0.492	0.675	0.497	0.624	0.617	0.553	0.591	0.664	

**u3k90**	Sea Australia	Sea Indonesi	Kalimantan3	Kalimantan2	Papua30	Papua27	Papua32	Papua1	Papua4	Papua5
Sea Australia		0.003	0.001	0.033	0.001	0.004	0.007	0.001	0.001	0.001
Sea Indonesia	0.45		0.001	0.048	0.002	0.011	0.007	0.003	0.001	0.001
Kalimantan3	0.62	0.421		0.006	0.001	0.001	0.001	0.001	0.001	0.001
Kalimantan2	0.4	0.162	0.191		0.019	0.037	*0.06*	0.009	0.008	0.007
Papua30	0.645	0.503	0.652	0.595		0.002	0.002	0.001	0.001	0.001
Papua27	0.663	0.547	0.716	0.691	0.737		0.011	0.002	0.001	0.001
Papua32	0.622	0.47	0.677	0.609	0.667	0.743		0.002	0.001	0.001
Papua1	0.643	0.489	0.608	0.595	0.665	0.747	0.699		0.001	0.001
Papua4	0.617	0.49	0.572	0.482	0.644	0.693	0.664	0.612		0.001
Papua5	0.616	0.436	0.573	0.502	0.568	0.636	0.587	0.508	0.546	

**u10k90**	Sea Australia	Sea Indonesi	Kalimantan3	Kalimantan2	Papua30	Papua27	Papua32	Papua1	Papua4	Papua5
Sea Australia		0.003	0.001	0.024	0.001	0.003	0.006	0.001	0.001	0.001
Sea Indonesia	0.475		0.001	*0.055*	0.001	0.006	0.008	0.001	0.001	0.001
Kalimantan3	0.626	0.425		0.006	0.001	0.001	0.001	0.001	0.001	0.001
Kalimantan2	0.474	0.218	0.139		0.017	0.05	*0.068*	0.01	0.011	0.005
Papua30	0.7	0.574	0.694	0.69		0.002	0.001	0.001	0.001	0.001
Papua27	0.7	0.559	0.707	0.753	0.78		0.009	0.001	0.001	0.001
Papua32	0.679	0.545	0.702	0.674	0.705	0.766		0.001	0.001	0.001
Papua1	0.685	0.534	0.661	0.599	0.69	0.74	0.707		0.001	0.001
Papua4	0.667	0.564	0.63	0.543	0.715	0.723	0.698	0.696		0.001
Papua5	0.638	0.451	0.616	0.487	0.591	0.6	0.579	0.551	0.624	

**u3k95**	Sea Australia	Sea Indonesi	Kalimantan3	Kalimantan2	Papua30	Papua27	Papua32	Papua1	Papua4	Papua5
Sea Australia		0.001	0.001	0.026	0.001	0.004	0.009	0.001	0.001	0.001
Sea Indonesia	0.424		0.001	*0.054*	0.001	0.004	0.009	0.001	0.001	0.001
Kalimantan3	0.617	0.428		0.002	0.001	0.001	0.001	0.001	0.001	0.001
Kalimantan2	0.365	0.15	0.194		0.022	*0.056*	*0.079*	0.011	0.005	0.007
Papua30	0.593	0.445	0.612	0.554		0.001	0.005	0.001	0.001	0.001
Papua27	0.629	0.498	0.723	0.677	0.702		0.009	0.001	0.001	0.001
Papua32	0.616	0.459	0.709	0.592	0.657	0.736		0.002	0.001	0.001
Papua1	0.602	0.416	0.556	0.556	0.589	0.722	0.699		0.001	0.001
Papua4	0.588	0.468	0.525	0.468	0.574	0.691	0.686	0.541		0.001
Papua5	0.602	0.428	0.528	0.506	0.524	0.659	0.651	0.43	0.478	

**u10k95**	Sea Australia	Sea Indonesi	Kalimantan3	Kalimantan2	Papua30	Papua27	Papua32	Papua1	Papua4	Papua5
Sea Australia		0.003	0.001	0.026	0.001	0.003	0.006	0.001	0.001	0.001
Sea Indonesia	0.473		0.001	0.049	0.001	0.009	0.01	0.004	0.001	0.001
Kalimantan3	0.629	0.387		0.006	0.001	0.001	0.001	0.001	0.001	0.001
Kalimantan2	0.445	0.19	0.155		0.02	*0.051*	*0.057*	0.014	0.004	0.008
Papua30	0.647	0.532	0.645	0.66		0.002	0.003	0.001	0.001	0.001
Papua27	0.691	0.559	0.716	0.745	0.753		0.008	0.002	0.001	0.001
Papua32	0.644	0.532	0.693	0.678	0.686	0.783		0.001	0.001	0.001
Papua1	0.628	0.446	0.567	0.536	0.61	0.713	0.696		0.001	0.001
Papua4	0.664	0.54	0.58	0.53	0.651	0.72	0.693	0.636		0.001
Papua5	0.616	0.423	0.52	0.433	0.534	0.603	0.608	0.439	0.561	

**u3k99**	Sea Australia	Sea Indonesi	Kalimantan3	Kalimantan2	Papua30	Papua27	Papua32	Papua1	Papua4	Papua5
Sea Australia		0.002	0.001	0.031	0.001	0.004	0.001	0.001	0.001	0.001
Sea Indonesia	0.31		0.001	*0.195*	0.001	0.008	0.014	0.001	0.001	0.001
Kalimantan3	0.565	0.449		0.004	0.001	0.001	0.001	0.001	0.001	0.001
Kalimantan2	0.272	0.098	0.122		0.033	0.036	*0.075*	0.019	0.006	0.008
Papua30	0.486	0.355	0.574	0.551		0.001	0.004	0.001	0.001	0.001
Papua27	0.521	0.412	0.721	0.646	0.663		0.004	0.001	0.001	0.001
Papua32	0.545	0.414	0.751	0.573	0.63	0.69		0.003	0.002	0.001
Papua1	0.491	0.378	0.535	0.604	0.537	0.71	0.728		0.001	0.001
Papua4	0.489	0.41	0.488	0.487	0.504	0.688	0.716	0.434		0.001
Papua5	0.551	0.437	0.538	0.612	0.526	0.696	0.731	0.507	0.444	

**u10k99**	Sea Australia	Sea Indonesi	Kalimantan3	Kalimantan2	Papua30	Papua27	Papua32	Papua1	Papua4	Papua5
Sea Australia		0.002	0.001	0.025	0.001	0.001	0.003	0.001	0.001	0.001
Sea Indonesia	0.432		0.001	*0.108*	0.002	0.01	0.01	0.001	0.001	0.001
Kalimantan3	0.678	0.432		0.005	0.001	0.001	0.001	0.001	0.001	0.001
Kalimantan2	0.402	0.136	0.185		0.026	0.043	*0.073*	0.007	0.004	0.005
Papua30	0.563	0.445	0.658	0.625		0.003	0.003	0.001	0.001	0.001
Papua27	0.615	0.487	0.727	0.692	0.711		0.009	0.001	0.001	0.001
Papua32	0.617	0.515	0.782	0.672	0.673	0.763		0.001	0.001	0.001
Papua1	0.548	0.328	0.521	0.463	0.545	0.665	0.72		0.001	0.001
Papua4	0.605	0.442	0.532	0.479	0.58	0.669	0.728	0.479		0.001
Papua5	0.595	0.387	0.507	0.409	0.53	0.575	0.683	0.36	0.468	

Supplemental Table 4: Estimates of genetic diversity (nucleotide diversity and heterozygosity) for *Suberites diversicolor* populations. Per filtering options, values are colored from low (red) to high (green) to visualize differences among genetic diversity index and filtering options.

Lake	Nucleotide Diversity 3X 30%	Nucleotide Diversity 3X 10%	Nucleotide Diversity 3X 5%	Nucleotide Diversity 3X 1%
Sea Australia	0.0095	0.0090	0.0090	0.0088
Sea Indonesia	0.0101	0.0097	0.0099	0.0095
Kalimantan3	0.0050	0.0051	0.0055	0.0058
Kalimantan2	0.0074	0.0072	0.0073	0.0080
Papua27	0.0037	0.0040	0.0044	0.0052
Papua30	0.0045	0.0047	0.0052	0.0069
Papua32	0.0053	0.0054	0.0059	0.0071
Papua1	0.0036	0.0039	0.0042	0.0045
Papua4	0.0047	0.0048	0.0052	0.0061
Papua5	0.0060	0.0060	0.0065	0.0065

Lake	Heterozygosity GL 3X 30%	Heterozygosity GL 3X 10%	Heterozygosity GL 3X 5%	Heterozygosity GL 3X 1%
Sea Australia	0.1420	0.1504	0.1562	0.1320
Sea Indonesia	0.1171	0.1281	0.1095	0.1140
Kalimantan3	0.0295	0.0323	0.0342	0.0479
Kalimantan2	0.1200	0.1145	0.1187	0.1212
Papua27	0.0211	0.0228	0.0273	0.0372
Papua30	0.0246	0.0233	0.0172	0.0240
Papua32	0.0451	0.0470	0.0492	0.0419
Papua1	0.0257	0.0226	0.0208	0.0221
Papua4	0.0235	0.0246	0.0185	0.0295
Papua5	0.0188	0.0120	0.0137	0.0091

Lake	Heterozygosity (He) GC 3x 30%	Heterozygosity (He) GC 3x 10%	Heterozygosity (He) GC 3x 5%	Heterozygosity (He) GC 3x 1%
Sea Australia	0.117	0.105	0.089	0.077
Sea Indonesia	0.157	0.119	0.117	0.125
Kalimantan3	0.081	0.043	0.027	0.014
Kalimantan2	0.034	0.024	0.023	0.019
Papua27	0.038	0.032	0.028	0.024
Papua30	0.052	0.037	0.029	0.019
Papua32	0.059	0.05	0.048	0.049
Papua1	0.054	0.03	0.021	0.01
Papua4	0.08	0.043	0.03	0.015
Papua5	0.095	0.043	0.027	0.011

Lake	Nucleotide Diversity 10X 30%	Nucleotide Diversity 10X 10%	Nucleotide Diversity 10X 5%	Nucleotide Diversity 10X 1%
Sea Australia	0.0098	0.0093	0.0086	0.0080
Sea Indonesia	0.0103	0.0095	0.0100	0.0100
Kalimantan3	0.0055	0.0056	0.0052	0.0057
Kalimantan2	0.0077	0.0072	0.0079	0.0080
Papua27	0.0048	0.0051	0.0073	0.0086
Papua30	0.0056	0.0061	0.0070	0.0091
Papua32	0.0062	0.0074	0.0081	0.0087
Papua1	0.0042	0.0043	0.0041	0.0037
Papua4	0.0052	0.0063	0.0074	0.0088
Papua5	0.0064	0.0059	0.0064	0.0060

Lake	Heterozygosity GL 10X 30%	Heterozygosity GL 10X 10%	Heterozygosity GL 10X 5%	Heterozygosity GL 10X 1%
Sea Australia	0.1469	0.1247	0.1452	0.1541
Sea Indonesia	0.1109	0.0955	0.0969	0.0893
Kalimantan3	0.0306	0.0426	0.0504	0.0536
Kalimantan2	0.1249	0.0970	0.1152	0.1117
Papua27	0.0253	0.0242	0.0309	0.0544
Papua30	0.0252	0.0249	0.0156	0.0381
Papua32	0.0426	0.0525	0.0391	0.0715
Papua1	0.0280	0.0212	0.0257	0.0357
Papua4	0.0245	0.0162	0.0237	0.0537
Papua5	0.0163	0.0194	0.0273	0.0080

Lake	Heterozygosity (He) GC 10x 30%	Heterozygosity (He) GC 10x 10%	Heterozygosity (He) GC 10x 5%	Heterozygosity (He) GC 10x 1%
Sea Australia	0.12	0.11	0.103	0.098
Sea Indonesia	0.143	0.13	0.112	0.104
Kalimantan3	0.079	0.068	0.044	0.02
Kalimantan2	0.044	0.038	0.035	0.026
Papua27	0.039	0.032	0.028	0.022
Papua30	0.055	0.047	0.038	0.026
Papua32	0.062	0.055	0.047	0.047
Papua1	0.057	0.047	0.036	0.022
Papua4	0.075	0.06	0.047	0.028
Papua5	0.092	0.071	0.049	0.03

Supplemental Table 6: Spearman correlations of genetic diversity indices nucleotide diversity and heterozygosity versus environmental and physical factors for *Suberites diversicolor* populations. Outcomes of different filtering options are displayed.

u3k70		
Nucleotide diversity ~	Spearman's rho	*p*-value
Temperature	-0.610	0.081
Salinity	0.368	0.330
Connection	0.433	0.250
Area	0.033	0.948

u10k70		
Nucleotide diversity ~	Spearman's rho	*p*-value
Temperature	-0.475	0.197
Salinity	0.452	0.222
Connection	0.433	0.250
Area	-0.067	0.880

u3k70		
Expected Heterozygosity ~	Spearman's rho	*p*-value
Temperature	-0.203	0.600
Salinity	0.243	0.529
Connection	0.533	0.148
Area	-0.067	0.880

u10k70		
Expected Heterozygosity ~	Spearman's rho	*p*-value
Temperature	-0.203	0.600
Salinity	0.159	0.683
Connection	0.533	0.148
Area	-0.217	0.581

u3k90		
Nucleotide diversity ~	Spearman's rho	*p*-value
Temperature	-0.610	0.081
Salinity	0.368	0.330
Connection	0.433	0.250
Area	0.033	0.948

u10k90		
Nucleotide diversity ~	Spearman's rho	*p*-value
Temperature	-0.356	0.347
Salinity	0.393	0.295
Connection	0.683	0.050
Area	-0.133	0.744

u3k90		
Expected Heterozygosity ~	Spearman's rho	*p*-value
Temperature	-0.276	0.472
Salinity	0.545	0.129
Connection	0.678	0.045
Area	0.458	0.215

u10k90		
Expected Heterozygosity ~	Spearman's rho	*p*-value
Temperature	-0.196	0.614
Salinity	0.197	0.611
Connection	0.577	0.104
Area	-0.251	0.515

u3k95		
Nucleotide diversity ~	Spearman's rho	*p*-value
Temperature	-0.576	0.104
Salinity	0.427	0.252
Connection	0.417	0.270
Area	0.017	0.982

u10k95		
Nucleotide diversity ~	Spearman's rho	*p*-value
Temperature	-0.509	0.162
Salinity	0.544	0.130
Connection	0.683	0.050
Area	-0.083	0.843

u3k95		
Expected Heterozygosity ~	Spearman's rho	*p*-value
Temperature	-0.213	0.583
Salinity	0.660	0.053
Connection	0.845	0.004
Area	-0.460	0.213

u10k95		
Expected Heterozygosity ~	Spearman's rho	*p*-value
Temperature	-0.102	0.794
Salinity	0.294	0.442
Connection	0.611	0.081
Area	-0.469	0.203

u3k99		
Nucleotide diversity ~	Spearman's rho	*p*-value
Temperature	-0.373	0.323
Salinity	0.444	0.232
Connection	0.567	0.121
Area	-0.083	0.843

u10k99		
Nucleotide diversity ~	Spearman's rho	*p*-value
Temperature	-0.102	0.795
Salinity	0.561	0.116
Connection	0.700	0.043
Area	-0.500	0.178

u3k99		
Expected Heterozygosity ~	Spearman's rho	*p*-value
Temperature	-0.545	0.129
Salinity	0.786	0.012
Connection	0.745	0.021
Area	-0.084	0.831

u10k99		
Expected Heterozygosity ~	Spearman's rho	*p*-value
Temperature	-0.128	0.742
Salinity	0.460	0.213
Connection	0.544	0.130
Area	-0.538	0.135

Genetic distance ~	3X 30%	3X 10%	3X 5%	3X 1%	10X 30%	10X 10%	10X 5%	10X 1%
Geographic distance	r = 0.007, p = 0.504	r = -0.067, p = 0.677	r = -0.071, p = 0.680	r = -0.084, p = 0.696	r = 0.066, p = 0.402	r = -0.024, p = 0.559	r = -0.045, p = 0.615	r = -0.039, p = 0.587
Environmental distance	r = 0.002, p = 0.503	r = -0.005, p = 0.495	r = -0.050, p = 0.557	r = -0.070, p = 0.609	r = 0.030, p = 0.404	r = -0.004, p = 0.503	r = -0.048, p = 0.571	r = -0.108, p = 0.662
Connection distance	r = 0.041, p = 0.441	r = 0.172, p = 0.286	r = 0.191, p = 0.264	r = 0.442, p = 0.047	r = 0.144, p = 0.193	r = 0.0585, p = 0.429	r = 0.006, p = 0.497	r = 0.111, p = 0.362

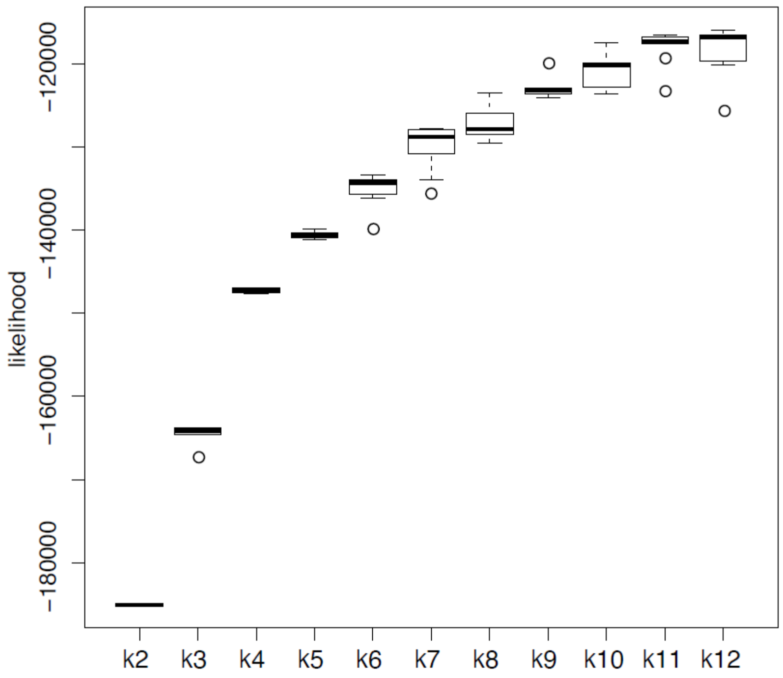

Supplemental Figure 1: Boxplot of likelihood values obtained from Admixture analyses for *Suberites diversicolor* populations. Values based on 10 replicate runs per putative ancestral population (K).

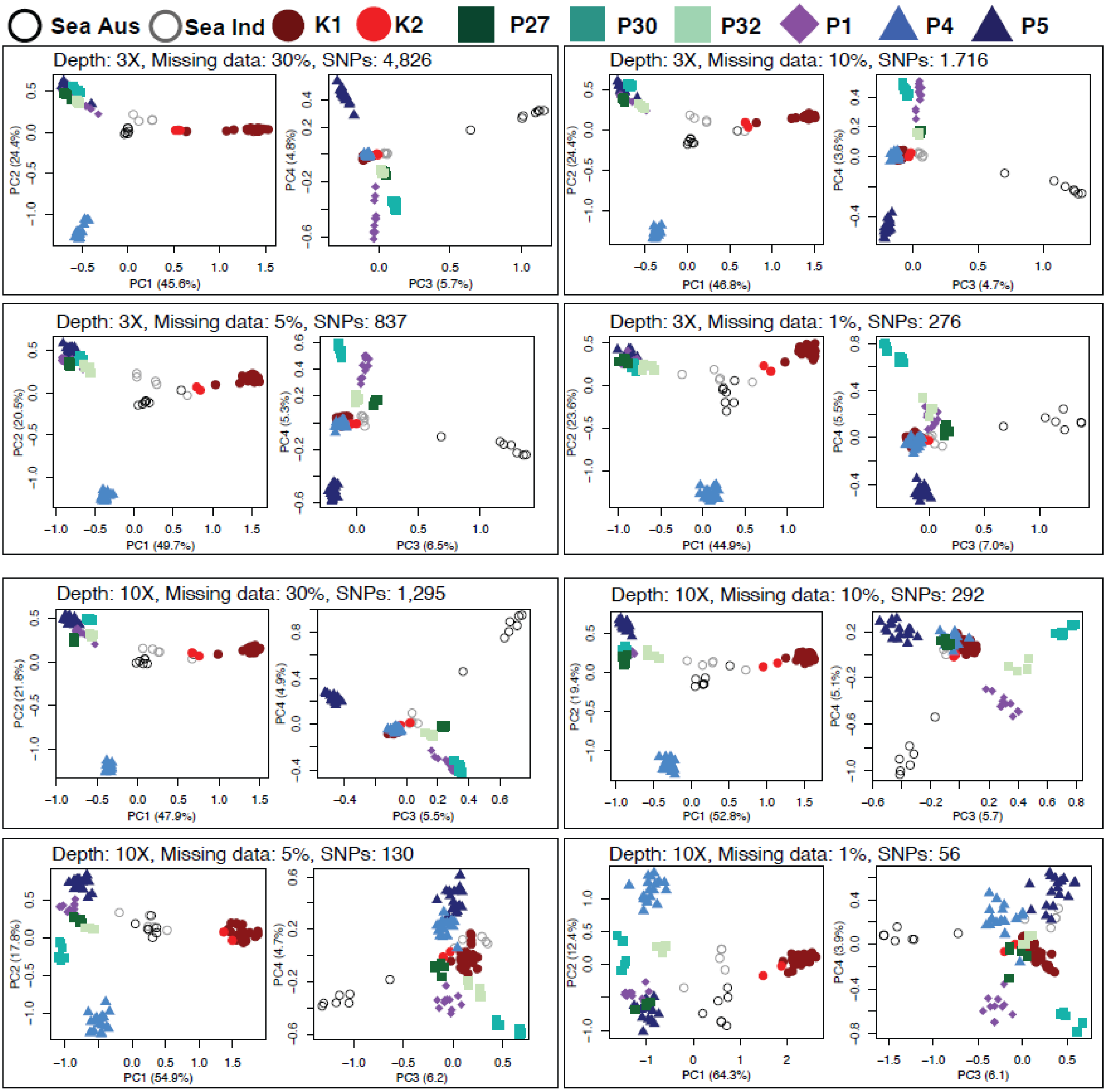

Supplemental Figure 2: Principal Component Analyses (PCA) for all different filtering options based on genotype likelihoods from *Suberites diversicolor* populations. Each dot represents one individual. Colors and codes correspond to Fig. 1 and Table 1.

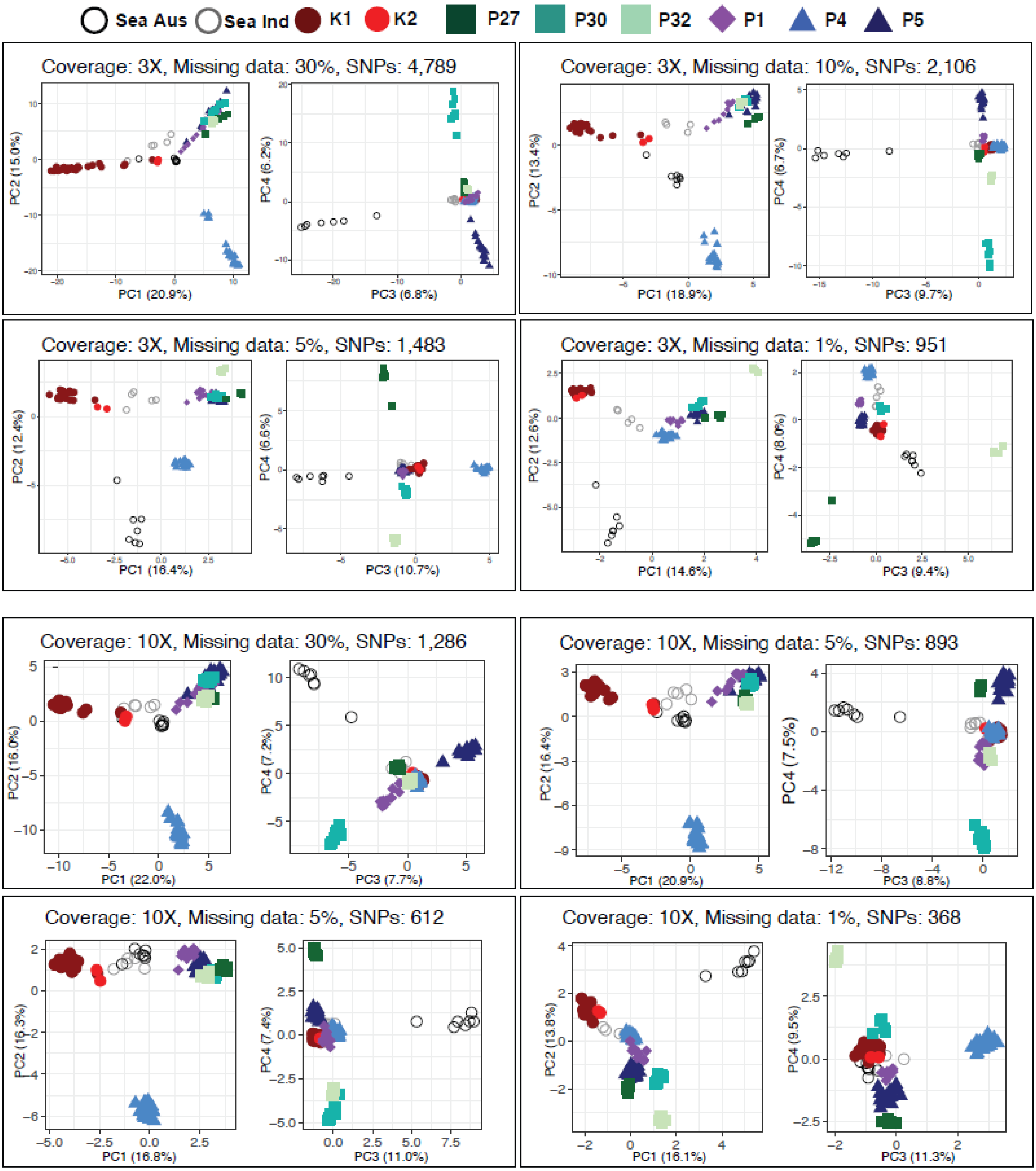

Supplemental Figure 3: Principal Component Analyses (PCA) for all different filtering options based on genotype calls from *Suberites diversicolor* populations. Each dot represents one individual. Colors and codes correspond to Fig. 1 and Table 1.

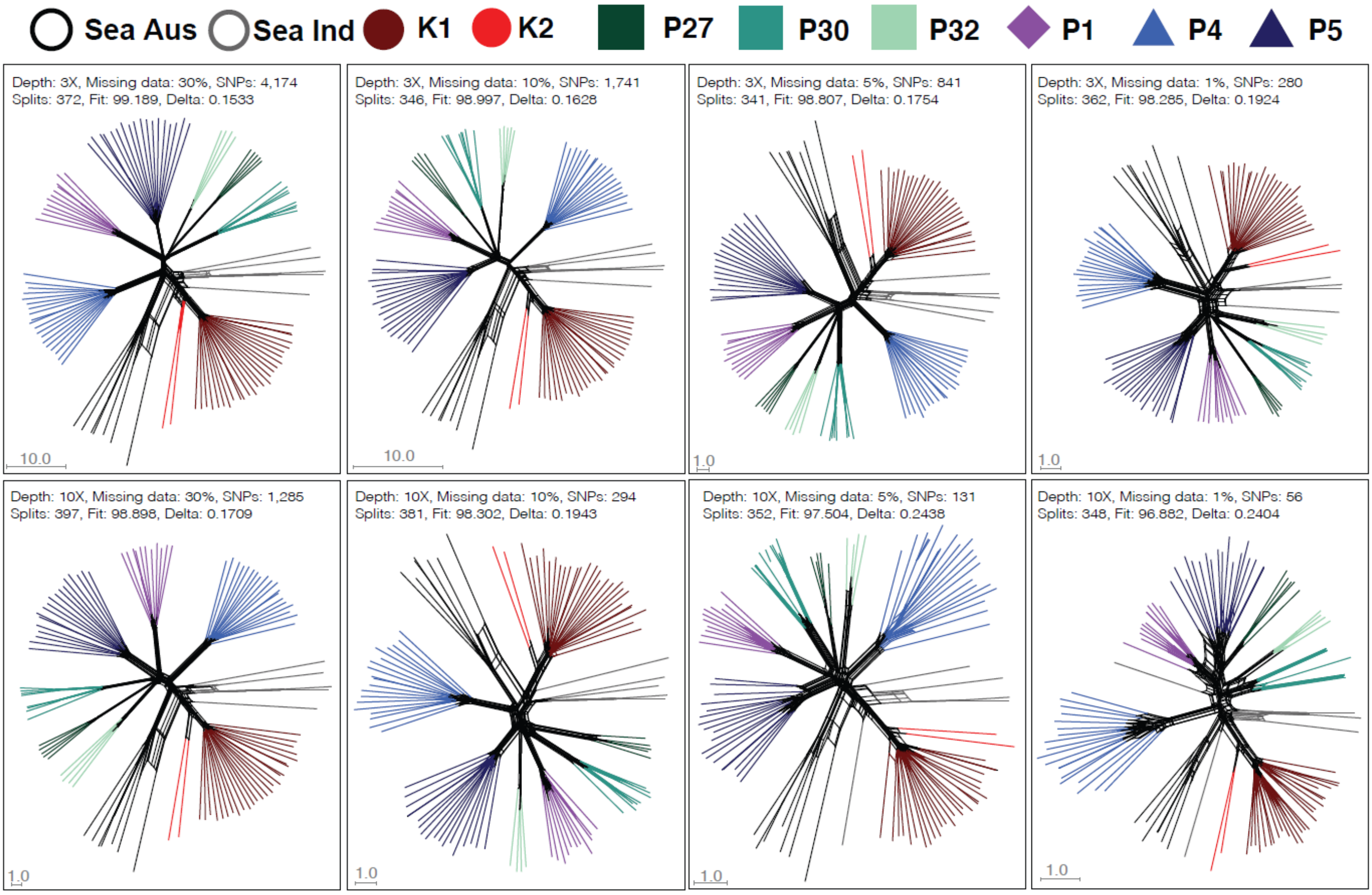

Supplemental Figure 4: Neighbor-Joining Network with equal angles computed in Splitstree based on pairwise genetic distances from different filtering options of *Suberites diversicolor* populations. Colors and codes correspond to Fig. 1 and Table 1.

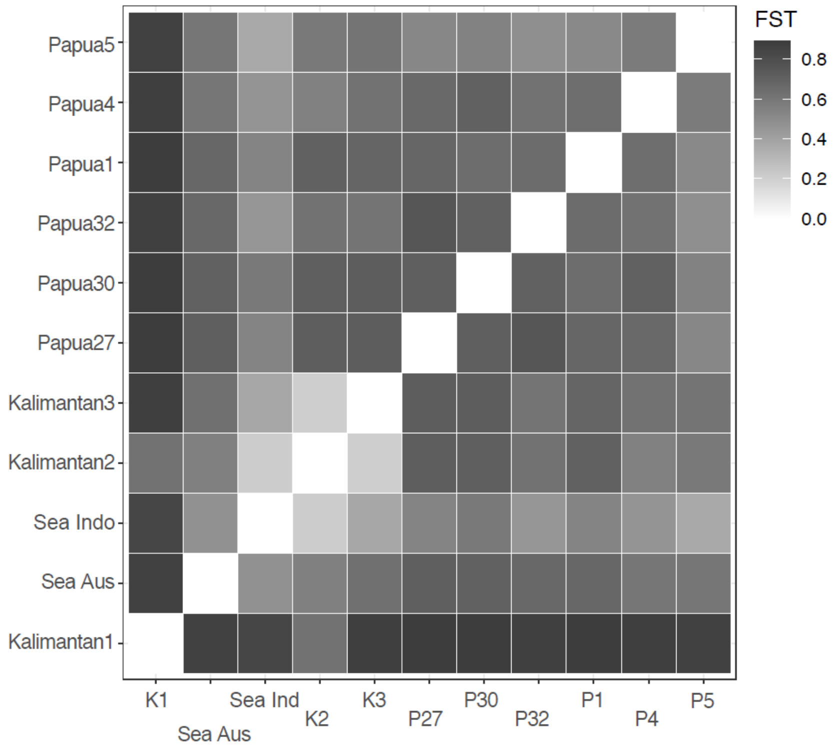

Supplemental Figure 5: Heatmap of normalized F'_{ST} values for populations of *Suberites diversicolor* including populations from Lineage A (Kalimantan1) and B (the rest).

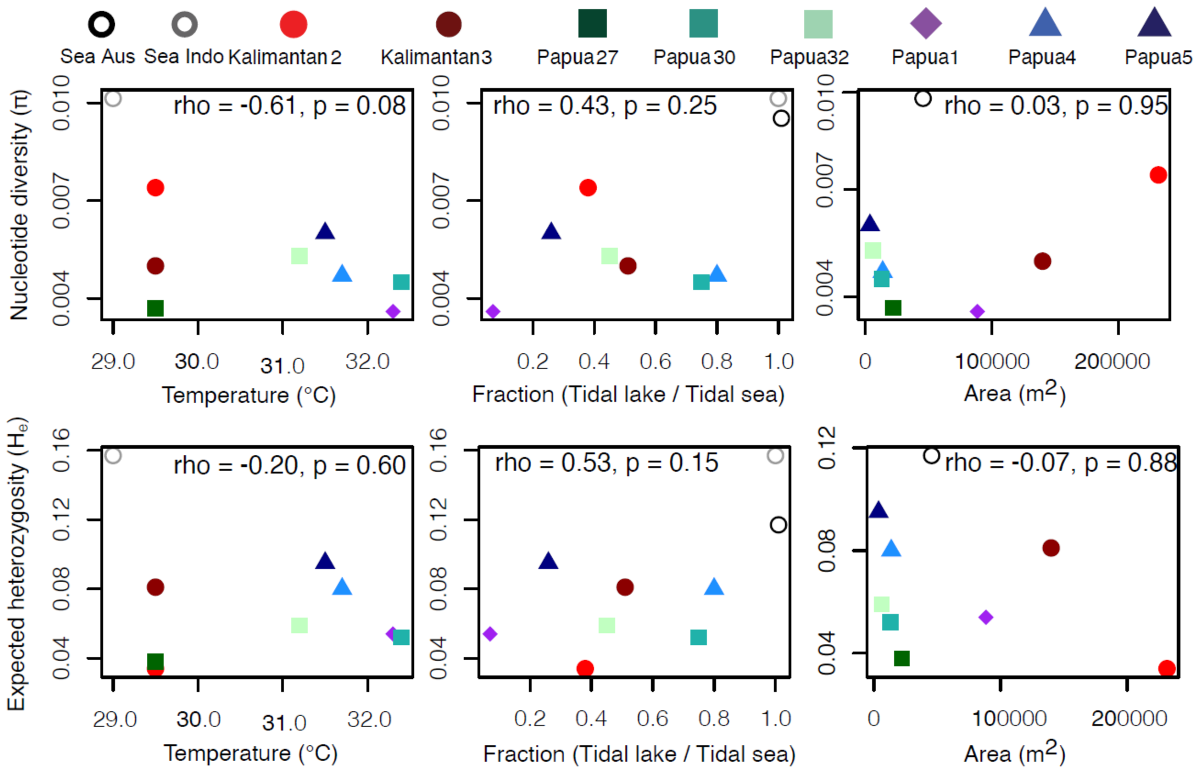

Supplemental Figure 6: Visualizations of Spearman correlations of genetic diversity indices nucleotide diversity and expected heterozygosity to environmental factors. Correlations to temperature, connection to the surrounding sea and lake area are displayed. Colors and codes correspond to Fig. 1 and Table 1.

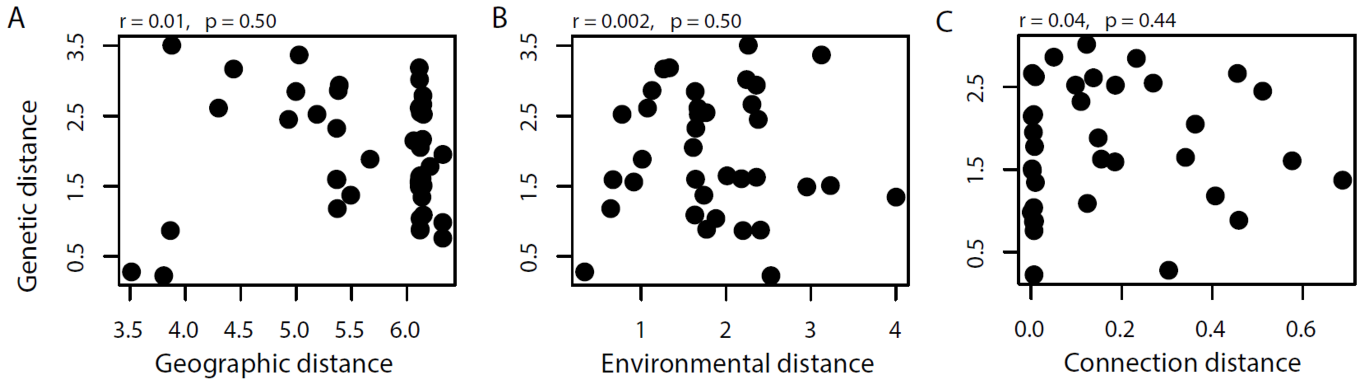

Supplemental Figure 7: Correlation tests (Mantel) between genetic distance matrix (F'_{ST}) versus A) geographic distance, B) environmental distance and C) connection distance for *Suberites diversicolor* populations.
