# Supplementary material for: Sponge diversification in marine lakes: Implications for phylogeography and population genomic studies on sponges
Source: Ecol Evol. 2023 Apr 13;13(4):e9945. doi: 10.1002/ece3.9945 (PMC10099488; doi:10.1002/ece3.9945)
Supplement: Supplementary file 2 — Appendix S1 [file ECE3-13-e9945-s001.pdf]

Supplemental Information for:

Sponge diversification in marine lakes: implications for phylogeography and population genomic studies on sponges

D.L. Maas, S. Prost, C.A. de Leeuw, K. Bi, L. Smith, Purwanto, L.P. Aji, R.F. Tapilatu, R. Gillespie, L.E. Becking

Table of Contents:

**S1: BioAnalyzer Restriction Enzymes**	Page 2
**S2: BioAnalyzer PippinPrep results**	Page 4

S1: BioAnalyzer Restriction Enzymes

Assay Class H gh Sens t v ty NA Assay Create 10/17/2014 4 31 55 PM
ata Path C.....gh Sens t v ty NA Assay 13804097 2014 10 17 16 31 56. a Mo f e 10/17/2014 6 17 12 PM

Electropherogram Summary Continued ...

S2

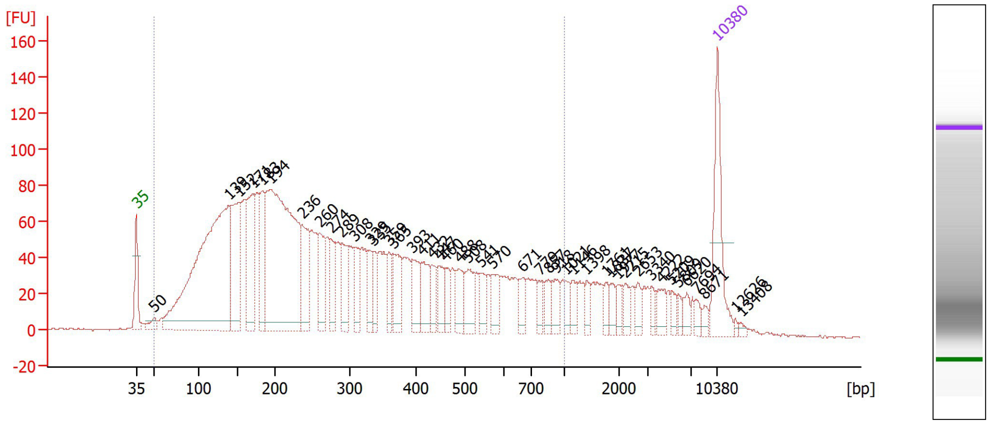

Overall Results for sample 2 **S2**

Number of peaks found: 45 Corr. Area 1: 3,462.3

Noise: 0.5

Peak table for sample 2 **S2**

Peak	Size [bp]	Conc. [pg/µl]	Molarity [pmol/l]	Observations	Area	Aligned Migration Time [s]	Peak Height	Peak Width	% of Total	Time corrected area
1	35	125.00	5,411.3	Lower Marker	29.0	43.00	63.5	1.0	0.0	66.5
2	50	15.85	480.5		6.9	45.22	6.3	1.4	0.5	15.1
3	139	616.53	6,735.5		339.7	54.13	69.6	8.1	21.1	609.8
4	152	152.85	1,525.5		87.6	55.33	71.1	1.2	5.3	153.7
5	171	139.06	1,232.1		85.3	57.08	75.7	1.1	5.0	144.9
6	183	78.18	648.7		49.9	58.15	77.6	0.6	2.9	83.2
7	194	490.88	3,839.2		325.5	59.16	78.6	4.3	18.4	532.3
8	236	88.04	564.4		65.7	62.99	59.2	1.1	3.5	100.6
9	260	63.94	373.3		50.6	65.07	54.8	0.9	2.6	74.9
10	274	45.90	253.4		37.7	66.41	51.3	0.7	1.9	54.7
11	289	53.83	281.8		45.9	67.75	49.2	0.9	2.3	65.1
12	308	41.86	206.2		37.3	69.32	47.2	0.7	1.8	51.7
13	329	31.05	142.8		29.2	71.07	45.4	0.6	1.4	39.4
14	335	30.22	136.7		28.8	71.54	44.6	0.6	1.3	38.6
15	359	28.12	118.6		28.4	73.47	44.5	0.6	1.3	37.0
16	365	49.87	206.8		51.1	73.98	43.3	1.1	2.3	66.1
17	393	43.60	167.9		47.6	76.25	40.7	1.1	2.1	59.7
18	411	40.98	151.2		45.8	77.40	39.1	1.1	2.0	56.6
19	432	27.72	97.2		31.5	78.65	37.9	0.8	1.3	38.3
20	447	30.07	102.0		34.6	79.48	37.1	0.9	1.4	41.6
21	460	23.02	75.9		26.8	80.22	36.2	0.7	1.1	31.9
22	488	33.64	104.4		40.0	81.88	35.5	1.1	1.6	46.6
23	508	38.11	113.6		46.3	82.94	35.5	1.2	1.8	53.3
24	541	24.50	68.7		31.3	84.46	33.5	0.9	1.2	35.3

Assay Class H gh Sens t v ty NA Assay Create 10/17/2014 4 31 55 PM
ata Path C..... gh Sens t v ty NA Assay 13804097 2014 10 17 16 31 56. a Mo f e 10/17/2014 6 17 12 PM

Electropherogram Summary Continued ...

... Peak table for sample 2 **S2**

Pea k	Size [bp]	Conc. [pg/µl]	Molarity [pmol/l]	Observati ons	Area	Aligned Migration Time [s]	Peak Height	Peak Width	% of Total	Time corrected area
25	570	26.66	70.9		35.6	85.85	32.6	1.0	1.4	39.5
26	671	21.01	47.5		29.1	89.54	31.2	0.9	1.1	31.0
27	779	15.17	29.5		22.0	91.58	30.1	0.7	0.8	22.9
28	857	17.53	31.0		26.6	92.64	30.7	0.8	0.9	27.4
29	918	22.59	37.3		35.6	93.47	30.5	1.1	1.3	36.2
30	1,021	13.19	19.6		21.8	94.72	30.2	0.7	0.8	21.9
31	1,146	14.53	19.2		24.7	95.55	29.8	0.8	0.8	24.5
32	1,398	12.89	14.0		23.0	97.21	29.9	0.7	0.8	22.4
33	1,761	10.38	8.9		19.8	99.61	29.1	0.6	0.7	18.9
34	1,837	12.32	10.2		23.9	100.12	28.9	0.8	0.8	22.6
35	1,977	11.79	9.0		23.4	101.04	28.0	0.8	0.8	22.0
36	2,215	13.03	8.9		26.2	101.92	28.7	0.9	0.8	24.3
37	2,653	12.08	6.9		24.6	103.40	29.4	0.8	0.8	22.5
38	3,340	8.87	4.0		18.3	105.01	27.4	0.6	0.6	16.5
39	4,222	15.11	5.4		31.3	106.17	25.8	1.2	1.0	27.9
40	5,209	10.12	2.9		21.0	107.46	25.0	0.8	0.6	18.5
41	5,809	6.76	1.8		14.1	108.24	23.2	0.6	0.4	12.3
42	6,620	10.66	2.4		22.3	109.31	24.2	1.0	0.7	19.3
43	7,694	8.11	1.6		17.4	110.46	20.2	0.9	0.5	14.9
44	8,671	6.44	1.1		14.2	111.38	17.0	0.8	0.3	12.1
45	10,380	75.00	10.9	Upper Marker	173.3	113.00	160.7	3.0	0.0	145.1
46	12,626	0.00	0.0		5.5	115.12	10.3	0.6	0.0	4.5
47	13,408	0.00	0.0		6.9	115.86	8.1	1.0	0.0	5.7

Region table for sample 2 **S2**

From [bp]	To [bp]	Corr. Area	% of Total	Average Size [bp]	Size distribution in C [%]	Conc. [pg/µl]	Molarity [pmol/l]	Co lor
50	1,000	3,462.3	88	322	61.2	3,008.39	23,670.9	

S2: BioAnalyzer PippinPrep results

2100 expert_High Sensitivity DNA Assay_DE13804097_2015-06-16_16-22-57.xad Page 10 of 11

Assay Class: High Sensitivity DNA Assay
Data Path: C:\...gh Sensitivity DNA Assay_DE13804097_2015-06-16_16-22-57.xad

Created: 6/16/2015 4:22:57 PM
Modified: 6/16/2015 6:23:58 PM

Electropherogram Summary Continued ...

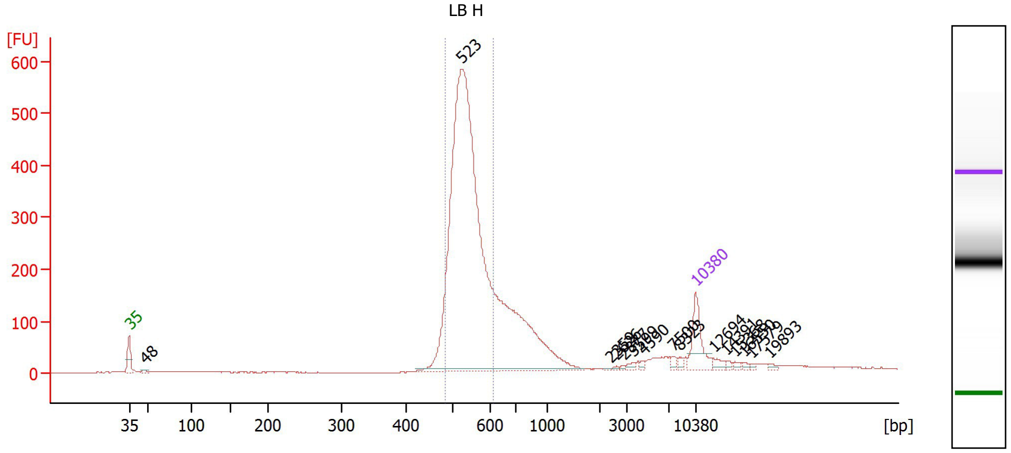

Overall Results for sample 4 **LB H**

Number of peaks found: 15 Corr. Area 1: 2,625.9

Noise: 0.6

Peak table for sample 4 **LB H**

Pea k	Size [bp]	Conc. [pg/µl]	Molarity [pmol/l]	Observati ons	Area	Aligned Migration Time [s]	Peak Height	Peak Width	% of Total	Time corrected area
1	35	125.00	5,411.3	Lower Marker	32.5	43.00	72.4	1.1	0.0	77.6
2	48	10.72	337.2		5.1	44.98	5.7	1.2	0.3	11.7
3	523	2,274.50	6,586.8		3,110.1	84.03	582.5	20.9	97.7	3,726.9
4	2,359	2.16	1.4		4.7	102.42	5.0	1.1	0.1	4.7
5	2,626	2.87	1.7		6.4	103.31	8.1	0.9	0.2	6.2
6	2,877	3.30	1.7		7.4	104.15	10.6	0.8	0.2	7.1
7	3,599	6.98	2.9		15.7	105.34	15.7	1.2	0.4	15.0
8	4,590	6.02	2.0		13.6	106.62	18.9	0.7	0.3	12.8
9	7,500	7.05	1.4		16.4	110.23	26.4	0.6	0.4	14.9
10	8,323	7.63	1.4		18.1	111.02	24.6	0.7	0.4	16.4
11	10,380	75.00	10.9	Upper Marker	189.0	113.00	148.8	3.1	0.0	167.6
12	12,694	0.00	0.0		35.0	115.22	23.1	1.8	0.0	30.4
13	14,391	0.00	0.0		11.8	116.86	16.5	0.7	0.0	10.1
14	15,368	0.00	0.0		13.9	117.80	14.9	1.0	0.0	11.8
15	16,550	0.00	0.0		11.9	118.93	13.4	0.9	0.0	10.0
16	17,579	0.00	0.0		9.6	119.92	11.8	0.8	0.0	8.0
17	19,893	0.00	0.0		11.4	122.15	10.6	1.2	0.0	9.3

Region table for sample 4 **LB H**

From [bp]	To [bp]	Corr. Area	% of Total	Average Size [bp]	Size distribution in C [%]	Conc. [pg/µl]	Molarity [pmol/l]	Co lor
486	612	2,625.9	67	537	5.7	1,581.37	4,469.8	

2100 Expert (B.02.08.S1648) © Copyright 2003 - 2009 Agilent Technologies, Inc. Printed: 6/16/2015 6:24:53 PM
