# Supplementary material for: Sponge diversification in marine lakes: Implications for phylogeography and population genomic studies on sponges
Source: Ecol Evol. 2023 Apr 13;13(4):e9945. doi: 10.1002/ece3.9945 (PMC10099488; doi:10.1002/ece3.9945)
Supplement: Supplementary file 3 — Appendix S2 [file ECE3-13-e9945-s002.pdf]

ddRADseq Protocol
Sponge diversification in marine lakes: implications for phylogeography and population genomic studies on sponges

Protocol based on: Peterson, Brant K., et al. "Double digest RADseq: an inexpensive method for de novo SNP discovery and genotyping in model and non-model species." *PloS one* 7.5 (2012): e37135
https://doi.org/10.1371/journal.pone.0037135

SeraMag bead dilution from: Rohland, Nadin, and David Reich. "Cost-effective, high-throughput DNA sequencing libraries for multiplexed target capture." *Genome research* 22.5 (2012): 939-946. doi:10.1101/gr.128124.111

IS4 and indexing oligos from: Meyer, Matthias, and Martin Kircher. "Illumina sequencing library preparation for highly multiplexed target capture and sequencing." *Cold Spring Harbor Protocols* 2010.6 (2010): pdb-prot5448. doi:10.1101/pdb.prot5448

ddRADseq TEST REACTIONS

ddRAD Ligation and PCR testing (Optional but highly recommended for anyone starting a ddRAD project for the first time.)

In addition to testing the adapters, this will also teach a researcher all steps of the protocol except for the Pippin Prep.

1) Double-digest a small subset of your samples in a 50 µL reaction according to the following protocol. Use samples for the testing where there is an abundance of material such that digestion can later be repeated if necessary.

Reagent	Volume (µL)	Final concentration
10x CutSmart Buffer	5.0	1x
MluCI (10,000U/mL)	1.5	0.2 U/µL
SphI-HF (100,000U/mL)	0.15	0.2 U/µL
DNA (600ng) + water	43.35	
Total volume	50	

2) Perform a 1.8x SPRI bead clean-up, following the protocol on page 6.

3) Set up a ligation reaction for each sample, using a different P1 adapter for each:

Reagent	Volume (µL)
T4 DNA Ligase Buffer*	4.0
400 units/µL T4 DNA Ligase*	0.5
P2-flex adapter dilution (10µM or less)*	2.0
P1-flex adapter dilution (10µM or less), unique for each sample	2.0
Double-digested bead-cleaned DNA + H_2O	31.5
Total volume	40

*The first 3 items can be made into a 6.2 µL master mix for easier and more accurate pipetting.
Note: if there is a white precipitate in the ligase buffer, warm it at 37°C until the solution becomes clear

4) On a thermocycler incubate at 23°C for 30 mins, heat kill at 65°C for 10 mins, then cool solution at 2°C per 90 seconds until it reaches 23°C. *(Note: this may need to be done on an iCycler in 4170 to allow for control over the final ramp time. If so, the setting is ramping from 65 to 23°C over 32 minutes)*

ddRADseq TEST REACTIONS

Temperature	Time	Cycles
23°C	30:00	x 1
65°C	10:00	x 1
Cool at 2°C per 90 seconds until the solution reaches a temperature of 23°C		

5) Perform a standard 1.8X SPRI bead clean-up by adding 60 µL of room-temperature SPRI bead solution and pipetting up and down to mix well. From step 2, follow the protocol on page 6 as written.

6) Qubit the post-ligation, post-clean-up product to have a baseline concentration value and to calculate how many µL is needed to use 20 ng in the subsequent PCR.

7) Set up a 20 µL PCR reaction for each ligation product:

Reagent	Volume (µL)
5x HF Phusion Buffer*	4.0
dNTPs (10 mM each)*	0.4
Phusion DNA polymerase (2U/µL)*	0.2
IS4 primer (10 µM)*	4
P7 indexing primer (10 µM)	4
Template DNA (30 ng/µL)	2
Nuclease-free H_2O	5.4

**The first 4 items can be made into a 7.4 µL master mix for easier and more accurate pipetting.*

8) Run the samples on a thermocycler under the following conditions:

Temperature	Time	Cycles
98°C	00:30	x 1
98°C	00:30	|
60°C	00:30	| x 12
72°C	01:00	|
72°C	05:00	x 1
4°C	∞	

ddRADseq TEST REACTIONS

9) Perform a standard 1.8X SPRI bead clean-up following the protocol on page 13.

10) Qubit the PCR product (post-clean-up) and calculate the total amount of DNA present. Compare with the amount added to the PCR reaction (~ 20 ng). If all components of the test libraries worked well, you should see significant growth in this value after PCR.

11) Run an agarose gel to check that the concentration change is <u>not</u> due solely to the growth of adapter dimer. You should see a smear of DNA that is brighter with small fragments and has a tail growing fainter towards the larger fragments. There should be very little product below 150 bp.

If you see a smear of DNA greater than 150 bp on the gel (see below), and if your total DNA values increased significantly before and after PCR, you have confirmed that everything is working fine. If adapters did not anneal properly to each other or ligate correctly to your digestion, you would not see any PCR amplification of your samples. If you do not see a result indicating that DNA amplified, do not start processing your samples until you figure out the cause.]

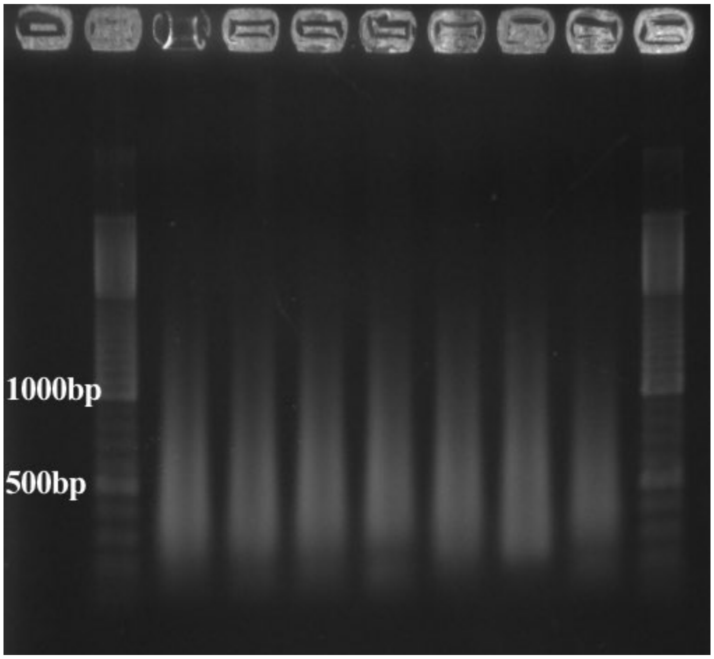

Gel of index PCR product from ddRAD ligation testing. Actual results may differ slightly.

[Note: Some adapter dimer present at ~135-140 bp is fine. But ensure that it is a small amount compared to your library smear.]

ddRADseq MAIN PROTOCOL

Double Digestion of Genomic DNA

As much as is possible, try to start this process using the same amount of genomic DNA for all of your project samples.

1) Quantify concentration from each sample using Qubit.

2) Calculate μL needed to start with 600 ng of high quality genomic DNA per sample.
Note: If some samples will have less starting materials than the threshold you choose, group them near to one another (in the same plate or strip tubes) during digestion. This will help make later sample organization much simpler.

3) Add the DNA to the appropriate wells of your plate. Add water such that the total volume of DNA + water is 43.5 μL.

4) Make the following master mix and add 6.5 μL to each sample:

Reagent	Volume (μL)	Final concentration
10x CutSmart Buffer	5	1X
MluCI (10,000 U/mL)	1.5	0.2 U/μL
SphI-HF (100,000 U/mL)	0.15	0.2 U/μL

5) Digest in a thermocycler for 3 hours at 37°C. Depending on the cycler, the following program might be necessary:

Temperature	Time	Cycles
37°C	60:00	} 3x
4°C	∞	

ddRADseq MAIN PROTOCOL

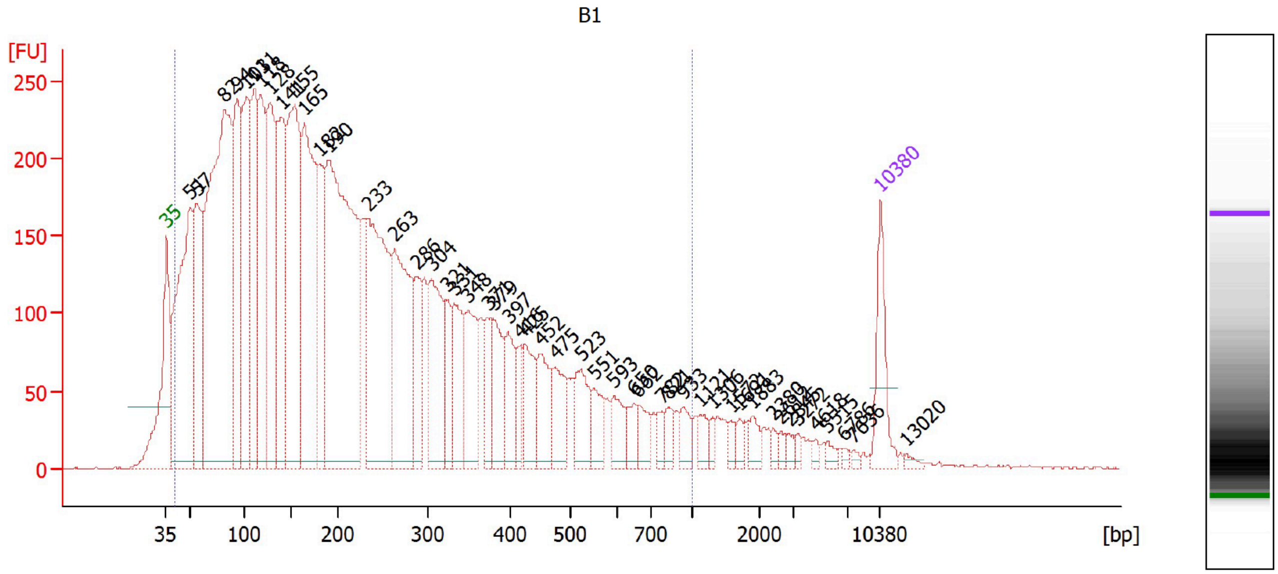

Overall Results for sample 5 : B1

Number of peaks found:	49	Corr. Area 1:	10,834.6
Noise:	0.6		

Post-digestion SPRI Bead Clean-up (1.5X)

IMPORTANT: Make sure beads warm up to room temp for 30 minutes before starting and are resuspended before use.

1) Add 75 µL of SPRI beads (Ampure/Kapa/diluted SeraMag from Rohland and Reich 2012) to each 50 µL sample. Mix thoroughly by pipetting up and down ~10 times and/or vortex gently for ~10 seconds on setting 4.

2) Incubate at room temperature for 5 minutes.

3) Spin briefly (0.5 second) to collect all liquid in bottom of tubes.

4) Place on magnetic stand for 5 minutes or until the liquid is completely clear.

5) Pipette out the clear solution (~125 µL) and discard.

6) Wash with 200 µL freshly made 80% EtOH, let sit for at least 30 seconds, then pipette out the liquid and discard.

7) Repeat step 6 once for a total of two washes.

8) Use a Rainin 20µL LTS pipette or other small volume tip to remove as much residual alcohol as possible without disturbing the beads. If necessary, used autoclaved toothpicks to remove small droplets of ethanol in the tube. Let dry for 3-5 minutes - be careful not to overdry (cracks appear in the beads).

9) Remove samples from magnetic plate and elute DNA in 33 µL EB or EBT. Mix by pipetting up and down or gently vortexing. Once homogenized, let sit at room temperature for 5 minutes to incubate.

10) Spin briefly (0.5 second) to collect all liquid in bottom of tubes.

11) Place samples back onto magnetic stand for 3 minutes or until the liquid is completely clear.

12) Remove supernatant (~30 µL) and place into a new, labeled tube, leaving beads behind.

ddRADseq MAIN PROTOCOL

Adapter Ligation

1) With the qubit values calculated on the previous page, make a spreadsheet indicating:
    a. The position or each sample
    b. The µL of digested product to add for 350 ng
    c. The µL of nuclease-free ddH2O to add to have a combined volume of 31.8 µL

2) In a new strip tube or PCR tube, add 350 ng of digested DNA and water to bring the volume to 31.5 µL

3) Add 2 µL of P1 Flex Adapter Dilution to each reaction (this attaches a unique internal barcode for each sample that will be pooled together in a set of 18). Using a multichannel pipette to add the adapters in sets of 8 greatly reduces the chance of an error.

4) Make the following master mix for all reactions:

Reagent	Volume (µL)	Final concentration
10 µM P2 Flex Adapter Dilution	2	
10X T4 DNA Ligase buffer^	4.0	1X
T4 DNA Ligase 400 U/µL	0.5	2.0 U/µL

^*Note: if there is a white precipitate in the ligase buffer, warm it at 37°C until the solution becomes clear*

5) Add 6.2 µL of the master mix to each reaction, and seal the tubes/plate well.

6) Incubate at 23°C for 30 minutes, heat kill at 65°C for 10 minutes, then cool solution 2°C per 90 seconds until it reaches 23°C. *(Note: this may need to be done on an iCycler in 4170 to allow for control over the final ramp time. If so, the setting is ramping from 65 to 23°C over 32 minutes)*

Temperature	Time	Cycles
23°C	30:00	x1
65°C	10:00	x1
Cool at 2°C per 90 seconds until the solution reaches a temperature of 23°C		

Post-Ligation SPRI Bead Clean-Up (1.5X)

IMPORTANT: Make sure SPRI beads warm up to room temp for 30 minutes before starting and are resuspended before use.

1) Put all libraries from each set into a 1.5mL tube. (In most cases this will be 18 x 40 µL reactions or 720 µL total volume.) Mix gently, then separate the 720 µL of pool into two 1.5mL tubes of 360 µL each

First 1.5x SPRI bead clean-up:

2) Add SPRI bead solution to each reaction as follows for a 1.5X clean-up:
   a) For each 360 µL pool, add 480 µL of room-temperature SPRI bead solution. Pipette up and down to mix.
   b) Seal the tube and gently vortex for several seconds.
   c) Let the tubes sit for 15 minutes at room temperature to incubate.
   d) Collect the liquid at the bottom of the wells by briefly centrifuging. Be careful not to centrifuge for more than a split second as the beads may collect in the bottom.

3) Place the tubes in a magnetic tube rack for and let sit for 5 minutes to separate the beads from solution. Pipette off and discard the supernatant without removing or disturbing the beads.

4) Leave on magnetic rack, and wash the beads by adding 1.5 mL of freshly prepared 80% ethanol. (You need to make a new 80% dilution before every clean-up). Let stand for 30 seconds and discard supernatant.

5) Repeat step 4.

6) Remove residual traces of ethanol using a small volume pipette and toothpicks to blot up any spots of ethanol. Let the beads air-dry for 5-10 minutes at room temperature without caps. DO NOT allow beads to over-dry, which appears as cracking.

7) Elute as follows after removing tubes from the magnetic rack:
   a) Add 50 µL of EB or EBT to the wells
   b) Resuspend the beads by pipetting up and down then sealing and gently vortexing.
   c) Let the tubes sit for 5 min at room temperature to incubate.
   d) Collect the liquid at the bottom of the wells by briefly centrifuging. Be careful not to centrifuge for more than a split second as the beads may collect in the bottom.

8) Place the tubes back on the magnetic rack, let stand for 3 minutes (or until liquid is completely clear), and transfer both 50 µL volumes of supernatant into the same new tube (strip tube or 1.5mL). Proceed to Second 1.5x SPRI bead clean-up on the following page.

Second 1.5x SPRI bead clean-up

9) Add SPRI bead solution to reaction as follows for a second 1.5X clean-up:
   a) For the 100 µL pool, add 150 µL of SPRI beads. Pipette up and down to mix.
   b) Seal the well and gently vortex for several seconds.
   c) Let the tubes sit for 15 minutes at room temperature to incubate.
   d) Collect the liquid at the bottom of the wells by briefly centrifuging. Be careful not to centrifuge for more than a split second as the beads may collect in the bottom.

10) Place the tubes in a magnetic stand and let sit for 5 minutes to separate the beads from solution. Pipette off and discard the supernatant without removing or disturbing the beads.

11) Leave on magnetic rack, and wash the beads by adding 300 µL of freshly prepared 80% ethanol. (You need to make a new dilution before every clean-up). Let stand for 1 min and discard supernatant.

12) Repeat step 11.

13) Remove residual traces of ethanol using a small volume pipette and toothpicks to blot up any spots of ethanol. Let the beads air-dry for 3-5 min at room temperature without caps. DO NOT allow beads to over-dry which appears as cracking.

14) Elute as follows <u>after removing tubes from the magnetic rack</u>:
   a) Add 60 µL EB or EBT to the wells.
   b) Resuspend the beads by pipetting up and down then sealing and gently vortexing.
   c) Let the tubes sit for 5 minutes at room temperature to incubate.
   d) Collect the liquid at the bottom of the wells by briefly centrifuging. Be careful not to centrifuge for more than a split second as the beads may collect in the bottom.

15) Place the tubes back on the magnetic rack, let stand for 3 minutes (or until liquid is completely clear), and transfer the supernatant into a new siliconized 1.5 mL tube.

16) Take a qubit value to know how much total material you are submitting.

17) Send 30 µL to the FGL for Pippin Prep (255 LSA), requesting a 476-576 gel excision window.

ddRADseq MAIN PROTOCOL

Indexing PCR

1) Initially, select just a few of your post-Pippen Prep samples for amplification. Then once you know the number of cycles used is sufficient to obtain enough material for sequencing (while not over-amplifying), then set up more reactions for all samples. The Peterson et al. 2012 protocol recommends 4-8 separate amplifications reactions for each library pool.

2) Make the following master mix for each 25 µL indexing PCR reaction:

Reagent	Volume (µL)
5x HF Phusion Buffer*	4.0
dNTPs (10 mM each)*	0.4
Phusion DNA polymerase (2U/µL)*	0.2
IS4 primer (10 µM)*	4.0
P7 indexing primer (10 µM), unique to each pool	4.0
≤ 20 ng of template DNA	2
Nuclease-free H_2O	5.4

**The first 4 items can be made into a 7.4 µL master mix for easier and more accurate pipetting.*

3) Run the samples on a thermocycler under the following conditions:

Temperature	Time	Cycles
98°C	00:30	
98°C	00:30	|
60°C	00:30	| x 8-12**
72°C	01:00	|
72°C	05:00	
4°C	∞	

4) Clean-up samples following the Post-PCR SPRI bead clean-up protocol on page 11. Multiple reactions from the same Pippin Prep pool/with the same index can be combined and cleaned together.

Post-PCR SPRI Bead Clean-Up (1.2X)

IMPORTANT: *Make sure beads warm up to room temp for 30 minutes before starting and are resuspended before use.*

1) For a single reaction clean-up, add 30 µL of SPRI beads (Ampure/Kapa/diluted SeraMag from Rohland and Reich 2012). Adjust this number to 1.2 times the combined reaction volume when pooling multiple reactions for clean-up.

2) Pipette up and down to mix and/or gently vortex once capped.

3) Collect the liquid at the bottom of the wells by briefly centrifuging. Be careful not to centrifuge for more than a couple of seconds as the beads may collect in the bottom.

4) Incubate at room temperature for 5 minutes.

5) Place on magnetic stand for 5 minutes to separate the beads from the solution.

6) Pipette off and discard the supernatant without removing or disturbing the beads.

7) While still on the magnet, wash beads with 200 µL freshly made 80% EtOH, let sit for at least 30 seconds, then pipette out and discard the supernatant.

8) Repeat step 7 once.

9) Remove residual traces of ethanol using a small volume pipette and toothpicks to blot up any spots of ethanol. Let the beads air-dry for 3-5 minutes at room temperature without caps. DO NOT allow beads to over-dry, which appears as cracking.

10) Remove samples from magnetic plate and elute DNA in 20 µL EB Buffer.

11) Pipette up and down to mix and/or gently vortex once capped.

12) Collect the liquid at the bottom of the wells by briefly centrifuging. Be careful not to centrifuge for more than a couple of seconds as the beads may collect in the bottom.

13) Let the tubes sit for 5 minutes at room temperature to incubate.

14) Place the tubes back on the magnetic rack, let stand for 3 minutes (or until liquid is completely clear). Remove supernatant (~20 µL) and place into a new labeled tube, leaving beads behind.

Note: if a second clean-up is needed to eliminate adapter dimer, resume the protocol from the beginning. (Add 24µL of SPRI beads to the supernatant collected in step 14).

ddRADseq MAIN PROTOCOL

Quality Control:

1) Split each final library into two siliconized tubes: 10 µL for QB3 submission tube and the remainder for QC and back-up.

2) Take a Qubit value for each final library from the QC tube.

3) Run a DNA 1000 bioanalyzer trace for each library from the QC tube. Verify that sizing is as expected (~ 50-60 bp larger than the post-Pippin Prep traces) and that little to no adapter dimer is seen around 135 bp.

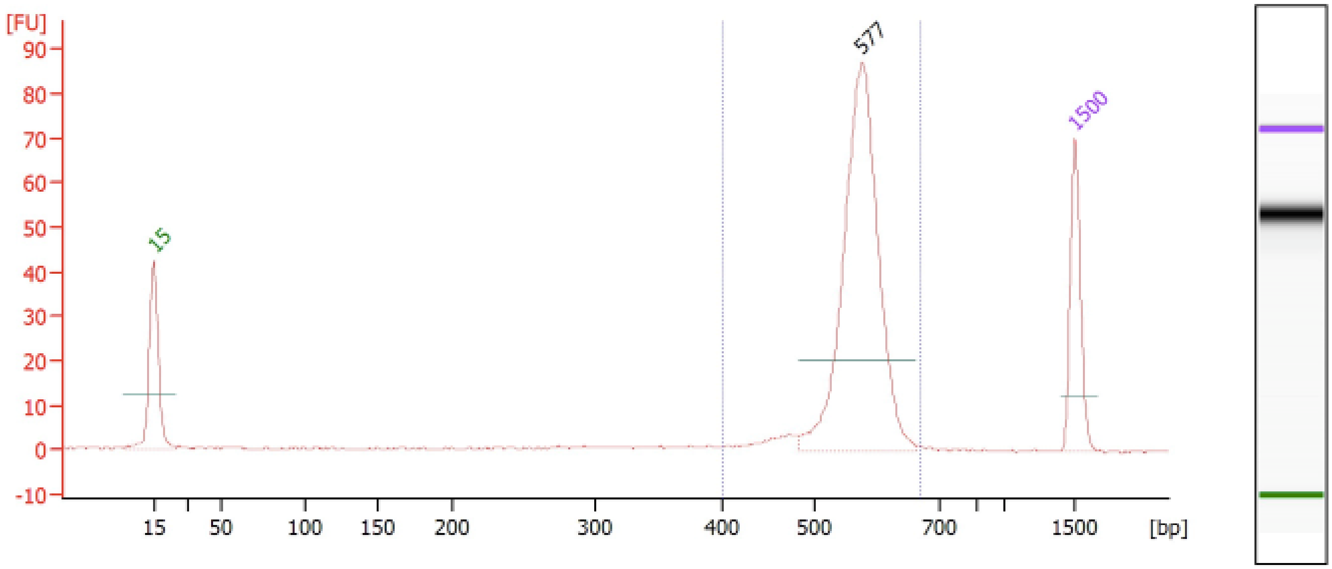

Overall Results for sample 4 : BL-4

Number of peaks found:	1	Area 1:	234.7

Peak table for sample 4 : BL-4

Peak		Size [bp]	Conc. [ng/µl]	Molarity [nmol/l]	Observations
1	◀	15	4.20	424.2	Lower Marker
2		577	10.53	27.7	
3	▶	1,500	2.10	2.1	Upper Marker

Region table for sample 4 : BL-4

From [bp]	To [bp]	Area	% of Total	Average Size [bp]	Size distribution in CV [%]	Conc. [ng/µl]	Color
400	670	234.7	84	567	7.1	11.40	

4) If adapter dimer is present, an additional bead clean-up may be required before sequencing submission. Otherwise samples may be submitted to QB3 for processing.
